# A metagenomics roadmap to the uncultured genome diversity in hypersaline soda lake sediments

**DOI:** 10.1186/s40168-018-0548-7

**Published:** 2018-09-19

**Authors:** Charlotte D. Vavourakis, Adrian-Stefan Andrei, Maliheh Mehrshad, Rohit Ghai, Dimitry Y. Sorokin, Gerard Muyzer

**Affiliations:** 1 Microbial Systems Ecology, Department of Freshwater and Marine Ecology, Institute for Biodiversity and Ecosystem Dynamics, Faculty of Science, University of Amsterdam, Postbus 94248, 1090 GE Amsterdam, the Netherlands; 2 Department of Aquatic Microbial Ecology, Institute of Hydrobiology, Biology Centre CAS, Na Sadkach 7, 370 05 Ceske Budejovice, Czech Republic; 3 Winogradsky Institute of Microbiology, Research Centre of Biotechnology, Russian Academy of Sciences, 60 let Oktyabrya pr-t, 7, bld. 2, Moscow, Russian Federation 117312; 4 Environmental Biotechnology, Department of Biotechnology, Delft University of Technology, Van der Maasweg 9, 2629 HZ Delft, the Netherlands

**Keywords:** Soda lake sediments, Metagenomics, Haloalkaliphilic extremophiles, Candidate Phyla Radiation, Wood-Ljungdahl pathway

## Abstract

**Background:**

Hypersaline soda lakes are characterized by extreme high soluble carbonate alkalinity. Despite the high pH and salt content, highly diverse microbial communities are known to be present in soda lake brines but the microbiome of soda lake sediments received much less attention of microbiologists. Here, we performed metagenomic sequencing on soda lake sediments to give the first extensive overview of the taxonomic diversity found in these complex, extreme environments and to gain novel physiological insights into the most abundant, uncultured prokaryote lineages.

**Results:**

We sequenced five metagenomes obtained from four surface sediments of Siberian soda lakes with a pH 10 and a salt content between 70 and 400 g L^−1^. The recovered 16S rRNA gene sequences were mostly from *Bacteria*, even in the salt-saturated lakes. Most OTUs were assigned to uncultured families. We reconstructed 871 metagenome-assembled genomes (MAGs) spanning more than 45 phyla and discovered the first extremophilic members of the Candidate Phyla Radiation (CPR). Five new species of CPR were among the most dominant community members. Novel dominant lineages were found within previously well-characterized functional groups involved in carbon, sulfur, and nitrogen cycling. Moreover, key enzymes of the Wood-Ljungdahl pathway were encoded within at least four bacterial phyla never previously associated with this ancient anaerobic pathway for carbon fixation and dissimilation, including the *Actinobacteria*.

**Conclusions:**

Our first sequencing effort of hypersaline soda lake sediment metagenomes led to two important advances. First, we showed the existence and obtained the first genomes of haloalkaliphilic members of the CPR and several hundred other novel prokaryote lineages. The soda lake CPR is a functionally diverse group, but the most abundant organisms in this study are likely fermenters with a possible role in primary carbon degradation. Second, we found evidence for the presence of the Wood-Ljungdahl pathway in many more taxonomic groups than those encompassing known homo-acetogens, sulfate-reducers, and methanogens. Since only few environmental metagenomics studies have targeted sediment microbial communities and never to this extent, we expect that our findings are relevant not only for the understanding of haloalkaline environments but can also be used to set targets for future studies on marine and freshwater sediments.

**Electronic supplementary material:**

The online version of this article (10.1186/s40168-018-0548-7) contains supplementary material, which is available to authorized users.

## Background

Soda lakes are evaporative, athallasic salt lakes with low calcium and magnesium concentrations and a high-alkaline pH up to 11 buffered by dissolved (bi-) carbonate ions [1]. They are constrained to arid regions across the globe, mainly the tropical East African Rift Valley [2], the Libyan Desert [3], the deserts in California and Nevada [4], and the dry steppe belt of Central Asia that spans to southern Siberia, north-eastern Mongolia, and Inner Mongolia in China [1]. On top of the extreme salinity and alkaline pH, the Eurasian soda lakes experience extreme seasonal temperature differences, causing highly unstable water regimes and fluctuating salinities [5]. Yet, soda lakes harbor diverse communities of haloalkaliphilic microbes, mostly prokaryotes that are well adapted to survive and grow in these extreme environments and consist of similar functional groups in soda lakes around the world [1, 2, 6]. The relative abundance of different groups is typically governed by the salinity of the brine [1, 7, 8], and microbial-mediated nutrient cycles become partially hampered only at salt-saturating conditions [1].

So far, all characterized prokaryotic lineages cultured from soda lakes comprise over 70 different species within more than 30 genera [1, 6, 9, 10]. From these, only a limited number of genomes have been sequenced today, mostly from chemolithoautotrophic sulfur-oxidizing bacteria belonging to the genus *Thioalkalivibrio* (class *Gammaproteobacteria*) [1, 11, 12]. It is well established that metagenomics enables the recovery of genomes and the identification of novel genetic diversity where culturing efforts fail [13, 14]. In recent years, next-generation sequencing has recovered a massive number of genomes from previously unknown groups of prokaryotes [15, 16], including a strikingly large and diverse group called “Candidate Phyla Radiation” (CPR), only distantly related to other cultured bacterial lineages [17]. Previously, we conducted a metagenomics study on soda lakes and reconstructed novel genomes from uncultured *Bacteroidetes* and “*Candidatus* Nanohaloarchaeaota” living in hypersaline Siberian soda brines [7]. Here, we turned our attention to the far more complex prokaryotic communities living in the sediments of the hypersaline soda lakes from the same region. We give a broad overview of all the taxonomic groups sequenced and focus on the metabolic diversity found in the reconstructed genomes of the most abundant, uncultured organisms.

## Results

### Overall prokaryote community structure

**Fig. 1:**
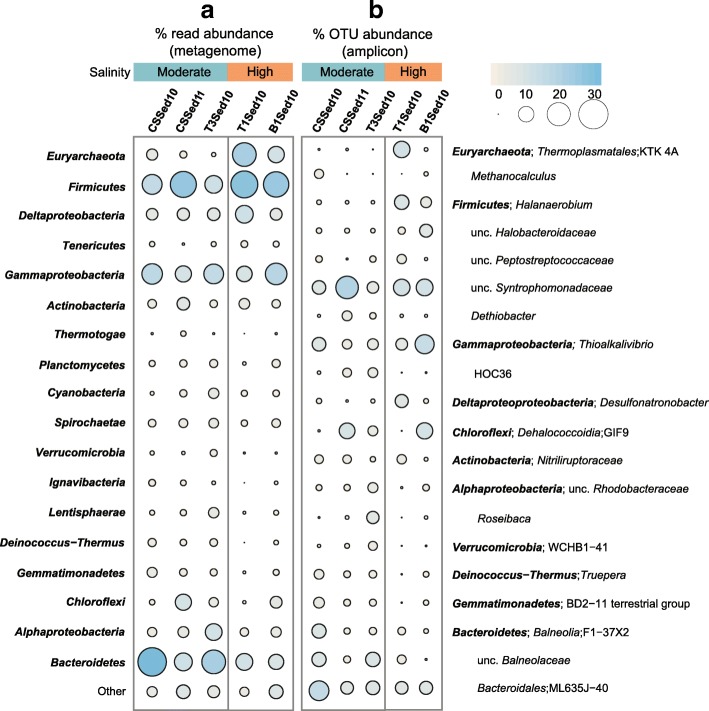
Abundant prokaryotic groups in five hypersaline soda lake sediments. **a** Relative abundance of the top-level taxa (those with ≥ 1% abundance in at least one dataset) based on 16S rRNA reads in unassembled metagenomic datasets. **b** Relative abundance of the 16S rRNA OTUs (those with sum of abundance in all datasets ≥ 3%) recovered by amplicon sequencing assigned where possible down to the genus-level. Three of the assessed soda lakes have a moderate salinity (70–110 g L^−1^), two are salt-saturated (400 g L^− 1^)

The salinities from the studied soda lakes ranged from moderately hypersaline (between 70 and 110 g L^−1^) to salt-saturated (400 g L^−1^ salt). The soluble carbonate alkalinity was in the molar range, and the pH in all lakes was around ten (see Additional file 1: Table S1). To give an overview of the overall prokaryotic community composition in each of the samples, we looked at the taxonomic classification of 16S rRNA genes recovered both by amplicon sequencing and direct metagenomics sequencing (Fig. 1, see also Additional file 2: Figure S1; Additional file 3). The prokaryotic communities of all five sediment samples were highly diverse and consisted mostly of uncultured taxonomic groups. *Bacteria* were more abundant than *Archaea*, regardless of the salinity of the overlaying brine [7] (Fig. 1). *Euryarchaeota* were the second and third largest group in the sediments of the two salt-saturated lakes comprising ~ 10 and ~ 20% of the 16S rRNA genes in the metagenomes. Most *Euryarchaeota*-related OTUs detected by amplicon sequencing belonged either to the uncultured *Thermoplasmata* group KTK 4A (SILVA classification) or the genera *Halohasta* and *Halorubrum* (class *Halobacteria*). In accordance with cultivation-dependent studies [6], most OTUs assigned to methanogens were from the class *Methanomicrobia*, especially the lithotrophic genus *Methanocalculus* (up to ~ 3%) and the methylotrophic genus *Methanosalsum* (Additional file 3)*.*

The varying ratio of the three dominant bacterial groups, *Firmicutes*, *Bacteroidetes* (including the newly proposed phyla *Rhodothermaeota* and *Balneolaeota* [18]), and *Gammaproteobacteria*, showed no clear trend in relation to the salinity in the lakes, but when *Firmicutes* were dominant, *Bacteroidetes* were less abundant and vice versa. Most *Firmicutes* belonged to the order *Clostridales*. Uncultured members from the family *Syntrophomonadaceae* had a relative abundance of more than 5% in all five metagenomes and comprised in two lakes even ~ 11–20% of the recovered amplicon sequences. The second most abundant *Firmicutes* order was *Halanaerobiales*, particularly the genus *Halanaerobium* (family *Halanaerobiaceae*) and uncultured members of the *Halobacteroidaceae*. The majority of *Bacteroidetes*-related OTUs could not be assigned down to the genus level. The uncultured ML635J-40 aquatic group (order *Bacteroidales*) comprised at least 5% of all five prokaryotic communities. This group has been previously found to be abundant in Mono Lake [4] (a soda lake) and in an anoxic bioreactor degrading cyanobacterial biomass under haloalkaline conditions [19]. Two other highly abundant (up to ~ 8%) uncultured groups from the class *Balneolia* (proposed new phylum *Balneolaeota* [18]) were also detected in other soda lakes before [3, 4]. Within the *Gammaproteobacteria*, the genus *Thioalkalivibrio* was abundant (~ 3% of the total community), but also uncultured members of HOC36 were prevailing at moderate salinities.

Members of the *Deltaproteobacteria*, *Alphaproteobacteria*, and *Chloroflexi* comprised up to ~ 10% of the detected 16S rRNA gene in some of the metagenomes. The GIF9 family of the class *Dehalococcoidia* was among the top three most abundant OTUs in two lakes. The extremely salt-tolerant and alkaliphilic genera *Desulfonatronobacter* (order *Desulfobacterales*) and *Desulfonatronospira* (order *Desulfovibrionales*) were the dominant *Deltaproteobacteria*. Highly abundant OTUs, within the *Actinobacteria* belonged to the class *Nitriliruptoria* and within the *Alphaproteobacteria* to the family *Rhodobacteraceae* and the genus *Roseibaca*. The important nitrifying genus *Nitrobacter* (*Alphaproteobacteria*) was present in only one of the lakes with moderate salinity (Additional file 3).

Some bacterial top-level taxa appeared less dominant (< 5%) from the 16S rRNA genes recovered from the metagenomes but were represented mainly by a single highly abundant OTU in the amplicon sequences, including the haloalkaliphilic genus *Truepera* within the phylum *Deinococcus*-*Thermus*, the genus *Spirochaeata* within the phylum *Spirochaetes*, the family BSN166 within the phylum *Ignavibacteriae*, the BD2–11 terrestrial group within the *Gemmatimonadetes*, and the WCHB1–41 order within the *Verrucomicrobia*. All OTUs within the *Thermotogae* and *Lentisphaerae* belonged to uncultured genera from the family *Kosmotogaceae* and *Oligosphaeraceae*, respectively. All *Tenericutes*-related OTUs belonged to the class *Mollicutes*, and especially the order NB1-n was dominant. In contrast, the phylum *Planctomycetes* was relatively diverse, with at least 11 different genus-level OTUs spread over four class-level groups.

### High-throughput genome recovery

**Fig. 2:**
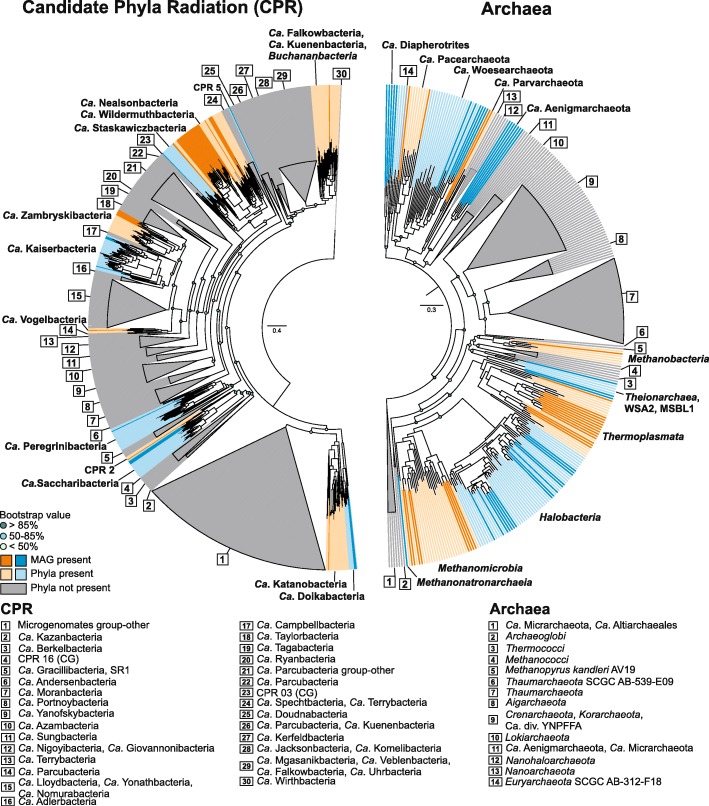
Maximum-likelihood phylogeny of the CPR and archaeal MAGs based on 16 ribosomal proteins. The archaeal tree is unrooted. The CPR tree is rooted to the *Wirthbacteria*. Alternate orange and blue colors show phyla/classes from which we obtained MAGs (labeled as “Phyla present”). Reconstructed MAGs of this study are highlighted by darker shades (labeled as “MAG present”). Phyla/classes for which there was no representative in the reconstructed MAGs of this study are shown as gray cartoons (labeled as “Phyla not present”), and the numerical labels are represented at the bottom. Colored circles at the nodes show confidence percentage of the bootstraps analysis (100×)

**Fig. 3:**
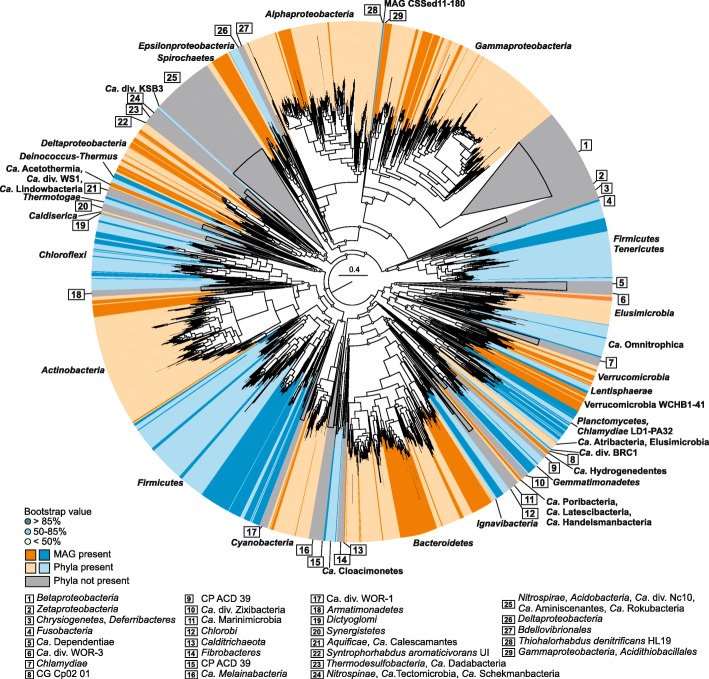
Maximum-likelihood phylogeny of the bacterial MAGs (CPR excluded) based on 16 ribosomal proteins. Alternate orange and blue colors show phyla/classes from which we obtained MAGs (labeled as “Phyla present”). Reconstructed MAGs of this study are highlighted by darker shades (labeled as “MAG present”). Phyla/classes for which there was no representative in the reconstructed MAGs of this study are shown as gray cartoons (labeled as “Phyla not present”), and the numerical labels are represented at the bottom. Colored circles at the nodes show confidence percentage of the bootstraps analysis (100×)

We obtained 717 medium-quality (≥ 50% complete, < 10% contamination) and 154 near-complete (≥ 90% complete, < 5% contamination) metagenome-assembled genomes (MAGs) across three major prokaryote groups: *Archaea*, *Bacteria*, and CPR (see Additional file 4 and Additional file 2: Figure S2). Figures 2 and 3 show the top-level phylogeny of all MAGs based on 16 ribosomal proteins. The reference database used contains a representative for each major prokaryote lineage [17]. We colored the different phyla from which we obtained a MAG in alternate blue and orange colors, and highlighted the MAGs obtained here in a darker shade. Many MAGs belonged to uncultured groups commonly detected in soda lake 16S rRNA gene surveys, over 100 MAGs still belonged to candidate prokaryote phyla and divisions that to our knowledge were never detected before in soda lakes, including CPR. Although only few MAGs had near-complete 16S rRNA genes, in most cases we were able to link available taxonomic gene annotations and ribosomal protein phylogeny to the SILVA taxonomy of the OTUs assigned to the amplicon sequences, while cross-checking the abundance profiles of both MAGs (Additional file 5) and OTUs.

The soda lake CPR recovered from the metagenomes was restricted to a few distinct phyla within the *Parcubacteria* group, mostly affiliating with “*Candidatus* Nealsonbacteria” and “Ca. Zambryskibacteria” [15] (Fig. 2). The first group of MAGs encompassed four different branches in our ribosomal protein tree, suggesting a high-phylogenetic diversity, with 33 putative new species sampled here (ANI and conDNA matrices given in Additional file 6). The “Ca. Zambryskibacteria-”related MAGs consisted of at least five new species. Few MAGs were recovered from CPR groups also detected by amplicon sequencing (see Additional file 2: Figure S1), namely the “Ca. Dojkabacteria” (former WS6), “Ca. Saccharibacteria” (former TM7), CPR2, and “Ca. Katanobacteria” (former WWE3).

Most archaeal MAGs belonged to the phylum *Euryarchaeota* and the abundant classes *Halobacteria*, *Methanomicrobia*, and *Thermoplasmata* (related to OTU KTK 4A) within. In addition, three *Thermoplasmata*-related MAGs that encoded for the key enzyme for methanogenesis (methyl-coenzyme M reductase, *mcr*) affiliated with reference genomes from *Methanomassilicoccales*, the seventh order of methanogens have been recovered [20, 21]. Another MCR-encoding MAG was closely related to the latest discovered group of poly-extremophilic, methyl-reducing methanogens from hypersaline lakes from the class *Methanonatronarchaeia* [9] (related to OTU ST-12K10A). We recovered also one MAG from the class *Methanobacteria* and a high-quality MAG from the WCHA1–57 group (“*Candidatus* Methanofastidiosa” [22]) in the candidate division WSA2 (Arc I). Several MAGs were recovered from the DPANN archaeal groups “Ca. Diapherotrites,” “Ca. Aenigmarchaeota,” (see Additional file 2: Figure S3) and “Ca. Woesearchaeota” (former Deep Sea Hydrothermal Vent Group 6, DHVEG-6). Although we did not reconstruct any reasonable-sized MAGs from the TACK superphylum, we found several 16S rRNA genes on the assembled contigs that affiliated to the *Thaumarchaeota* (see Additional file 1: Table S2).

Nearly every known bacterial phylum had an extremophilic lineage sampled from our hypersaline soda lake sediments (Fig. 3). In most cases, the soda lake lineages clearly formed separate branches appearing as sister groups to known reference lineages. The highest genome recovery was from the same top-level taxonomic groups that were also abundant in our 16S rRNA gene analysis. From the *Verrucomicrobia*, most MAGs belonged to the order WCHB1-41 (16S rRNA gene identity 92–100%). However, in our ribosomal protein tree, they branched within the phylum *Lentisphaerae*. Sixteen *Tenericutes* MAGs from at least 12 different species (Additional file 6) were closely related to the NB1-n group of *Mollicutes*. Based on the recovered genome size and encoded metabolic potential, these organisms are free-living anaerobic fermenters of simple sugars, similar to what has recently been proposed for “*Candidatus* Izimaplasma” [23]. Several MAGs belonged to the candidate phyla “Ca. Omnitrophica,” “Ca. Atribacteria,” and “Ca. Acetothermia” (former OP1), which were moderately abundant also in some sediment (see Additional file 2: Figure S1). For the latter phylum, we suspect that four MAGs were more closely related to ca. *div*. WS1 and “Ca. Lindowbacteria” for which only few reference genomes are currently available in NCBI (see Additional file 2: Figure S4). Due to a high-sequencing coverage, we also managed to reconstruct several MAGs from rare *Bacteria* (< 100 amplicon sequences detected, see Additional file 2: Figure S1), including the phyla “Ca. Hydrogenedentes,” “Ca. Cloacimonetes,” ca. div. BRC1, *Elusimicrobia*, *Caldiserica*, and “Ca. Latescibacteria.” The MAGs from the latter phylum were more closely related to the recently proposed phylum “Ca. Handelsmanbacteria” [15]. Two additional MAGs with 16S rRNA gene fragments with 94–95% identity to the class MD2898-B26 (*Nitrospinae*) were more likely members of ca. div. KSB3 (proposed “Ca. Moduliflexus” [24], see Additional file 2: Figure S5).

### Draft genomes of haloalkaliphilic CPR

**Fig. 4:**
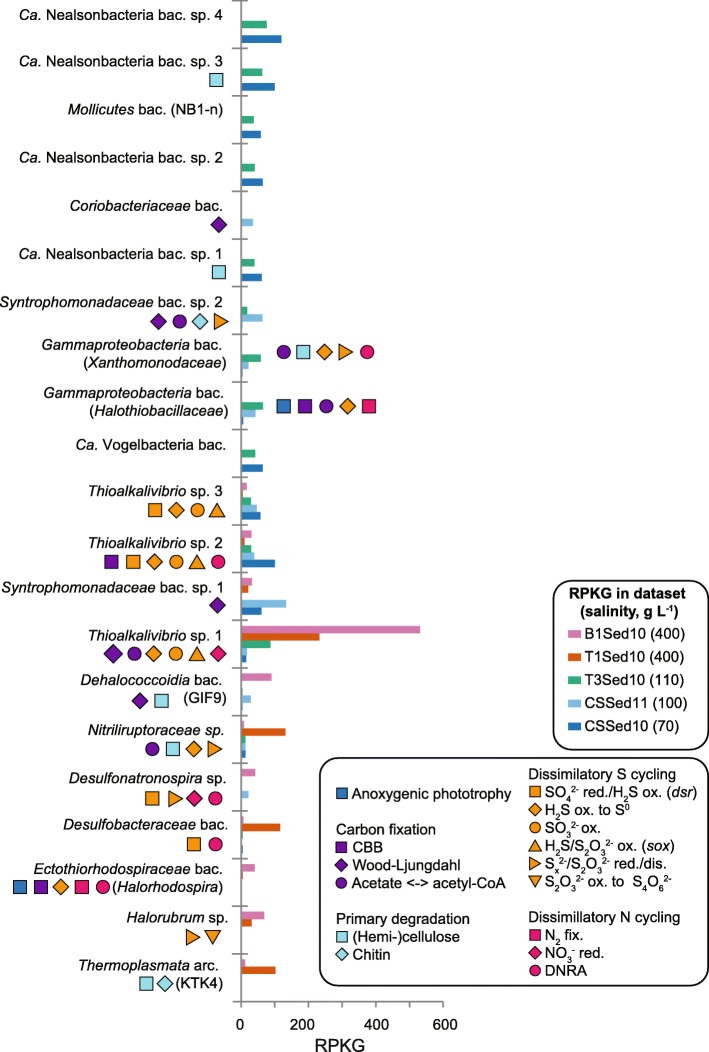
Relative abundance and metabolic potential of the dominant species. Abundance values, expressed as reads per kilobase of MAG per gigabase of mapped reads (RPKG), were averaged for the top ten abundant MAGs from each dataset that were (likely) the same species (Additional file 5, Additional file 6). Population genomes were ranked by their “salinity preference scores”: those recruiting relatively more from moderate salinity datasets (cold colors) are drawn to the top, from high salinity datasets (warm colors) to the bottom. The metabolic potential derived from functional marker genes (Additional file 7) is depicted by the colored symbols. CBB = Calvin-Benson-Bassham cycle, DNRA = dissimilatory nitrite reduction to ammonia, fix. = fixation, red. = reduction, ox. = oxidation, dis. = disproportionation

Strikingly, members of the CPR related to “Ca. Nealsonbacteria” and “Ca. Vogelbacteria” were among the top 5% of abundant organisms in the surface sediments of the soda lakes, especially those with moderate salinity (Fig. 4). Like most members of the CPR, the MAGs of the four most abundant “Ca. Nealsonbacteria” seem to be anaerobic fermenters [25]. They lacked a complete TCA cycle and most complexes from the oxidative electron transfer chain, except for the subunit F of a NADH-quinone oxidoreductase (complex I, *nuoF*, *nuoG, nuoA*) and *coxB* genes (complex II). All CPR MAGs had a near-complete glycolysis pathway (Embden-Meyerhof-Parnas) encoded, but pentose phosphate pathways were severely truncated. The commonly encoded F- and V-type ATPase can establish a membrane potential for symporter-antiporters by utilizing the ATP formed by substrate-level phosphorylation during fermentation. All CPR have V-type ATPases that can translocate Na^+^ in addition to H^+^ (see Additional file 2: Figure S6), while only two members of the “Ca. Falkowbacteria” had putative Na^+^-coupled F-type ATPases (see Additional file 2: Figure S7). The coupling of ATP hydrolysis to sodium translocation is advantageous to maintain pH homeostasis in alkaline environments. Interestingly, with only two exceptions [26, 27], all CPR genomes recovered from other environments with neutral pH were reported to encode only F-type ATPases [28–32]. One low-abundant MAG affiliated to “Ca. Peregrinibacteria” contained both the large subunit of a RuBisCO (type II/III, see Additional file 2: Figure S8) and a putative phosphoribulokinase (PRK, K00855) encoded in the same contig. This is remarkable because PRK homologs were not previously identified among CPR, and RuBisCo form II/III was inferred to function in a nucleoside salvage pathway [33]. One “Ca. Saccharibacteria” MAG encoded for a putative channelrhodopsin (see Additional file 2: Figure S9). This is the first rhodopsin found among the CPR and suggests that this enigmatic group of organisms may have acquired evolutionary adaptations to a life in sunlit surface environments.

**Fig. 5:**
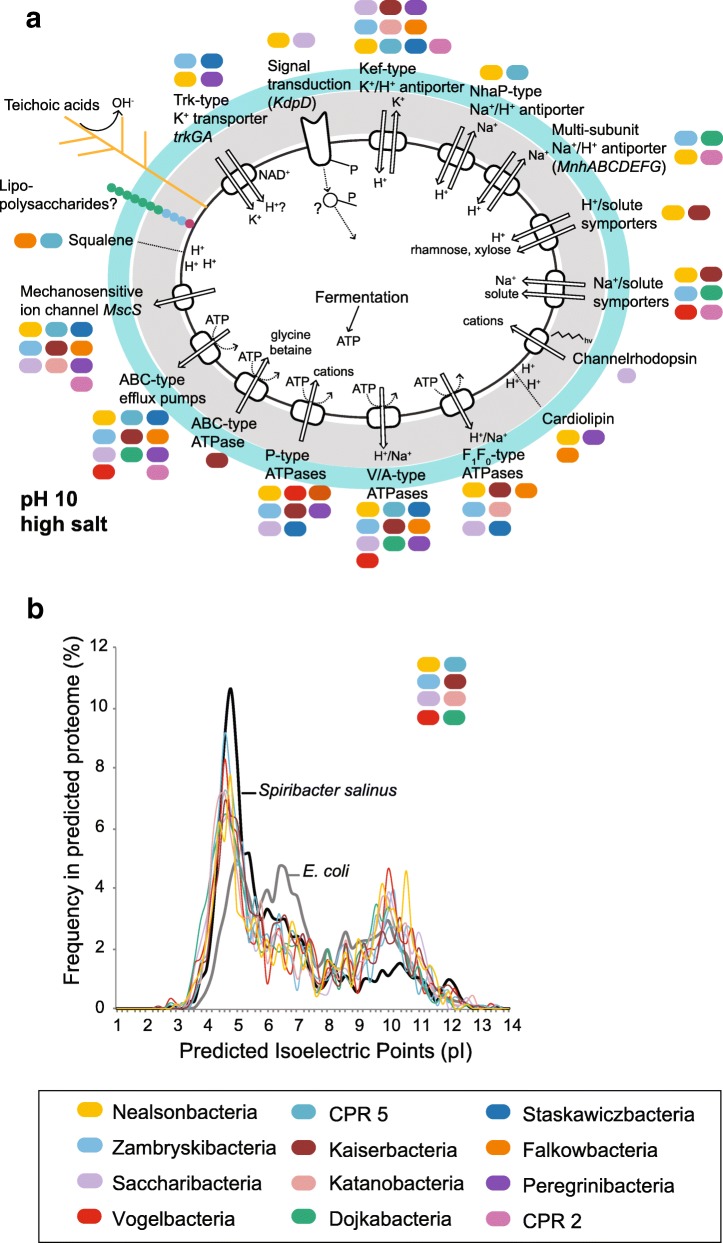
Potential mechanisms for regulating the intracellular pH and cytoplasmic ion content in different CPR phyla. **a** Membrane transporters, channels, and lipids. Peptidoglycan is depicted in gray and S-layer proteins in cyan. **b** Predicted isoelectric points (bin width 0.2) for the coding sequences of MAGs. A representative proteome is depicted for each phylum for which several members had a pronounced acidic peak (see also Additional file 2: Figure S11)

A previous study showed that most CPR has coccoid cell morphotypes with a monoderm cell envelope resembling those from Gram-positives and *Archaea* but with a distinct S-layer [34]. Thick peptidoglycans coated with acidic surface polymers such as teichoic acids help protect the cells of Gram-positives against reactive hydroxyl ions in highly alkaline environments [35] (Fig. 5a). All soda lake CPR had indeed the capability for peptidoglycan biosynthesis, but we found proteins typical for Gram-negatives for the biosynthesis of lipopolysaccharides (see Additional file 1: Table S3), homologous to the inner membrane proteins of type II secretion systems and to several proteins associated to the outer membrane and peptidoglycan, including *OmpA*.

It remains to be determined whether the soda lake CPR also lacks an outer membrane and perhaps anchor lipopolysaccharides, S-layer proteins, and lipoproteins to the inner cell membrane or peptidoglycan. We also found gene encoding cardiolipin and squalene synthases. Increased levels of cardiolipin and the presence of squalene make the cytoplasmic membrane less leaky for protons [36]. In addition, cation/proton exchangers are known to play a crucial role for pH homeostasis in alkaliphilic prokaryotes as they help acidify the cytoplasm during the extrusion of cations [35]. Putative Na^+^/H^+^ exchangers of the Nha-type and multi-subunit Mnh-type were found only within a few soda lake CPR. Secondary active transport of K^+^ might be mediated in most soda lake CPR by KefB (COG0475)/kch Kef-type, glutathione-dependent K^+^ transport systems, with or without H^+^ antiport (67,68).

Various soda lake CPR had an acidic proteome, with pI curves resembling those found in extremely halophilic *Bacteria*. Intracellular proteins enriched in acidic amino acids might be an adaptation to a “salt-in” strategy, i.e., maintaining high intracellular potassium (K^+^) concentrations to keep osmotic balance [7, 37] (Fig. 5b; see Additional file 2: Figure S10). Such a strategy is energetically favorable over de novo synthesis or import of osmolytes such as ectoine and glycine betaine. We did not find genes for the synthesis of organic osmolytes and homologs of ABC-type transporters for primary active uptake of proline/glycine betaine which were encoded only in one MAG (Fig. 5a). For the “Ca. *Nealsonbacteria*” and “Ca. *Vogelbacteria*,” the salt-in strategy might be a unique feature for the soda lake species explaining their high abundance in the hypersaline soda lake sediments, as we did not found an acidic proteome predicted from genomes obtained from other non-saline environments (See Additional file 2: Figure S11). The uptake of K^+^ ions remains enigmatic for most soda lake CPR. Low-affinity Trk-type K^+^ uptake transporters (generally with symport of H^+^) (67,68) were encoded only by a limited number of MAGs. We found three MAGs encoding for Kdp-type sensor kinases (*kdpD*) but no corresponding genes for the response regulator (*kdpE*) or for Kdp-ATPases that function as the inducible, high-affinity K^+^ transporters in other *Bacteria* (67,68). Finally, mechanosensitive ion channels (*mscS*, *mscL*) and ABC-type multidrug transport systems (*AcrAB*, *ccmA*, *EmrA*, *MdlB*, *NorM*) and sodium efflux permeases (*NatB*) were encoded in almost every MAG. The first might rapidly restore the turgor pressure under fluctuating salinity conditions by releasing cytoplasmic ions [38].

### Novel abundant groups involved in sulfur, nitrogen, and carbon cycles

A new species of *Thioalkalivibrio* (family *Ectothiorhodospiraceae*) was by far the most abundant in the sediments of the two salt-saturated lakes (Fig. 4). In the sediment of Bitter-1, also a purple sulfur bacterium from the same family was highly abundant. It was closely related to *Halorhodospira*, a genus also frequently cultured from hypersaline soda lakes [1]. None of the abundant *Ectothiorhodospiraceae* spp. had already a species-representative genome sequenced (Additional file 6). The potential of the *Thioalkalivibrio* spp. for chemolithotrophic sulfur oxidation was evident (Additional file 7; see Additional file 8: Information S1). Interestingly, the encoded nitrogen metabolisms were quite versatile. While *Thioalkalivibrio* sp. 1 had the potential for nitrate reduction to nitrite, *Thioalkalivibrio* sp. 2 might perform dissimilatory nitrite reduction to ammonia (DNRA; see Additional file 2: Figure S12).

Two deltaproteobacterial lineages of dissimilatory sulfate-reducing bacteria (SRB) were highly abundant in the soda lake sediment of Bitter-1. One MAG from the family *Desulfobacteraceae* (order *Desulfobacterales*) is the first genome from the genus *Desulfonatronobacter*. It encodes the genes for complete sulfate reduction to sulfide using various electron donors, as well as for the complete oxidation of volatile fatty acids and alcohols, a unique feature for the genus *Desulfonatronobacter* among haloalkaliphilic SRB [10] (see Additional file 8: Information S2). Fumarate and nitrite (DNRA, *NrfAH*) could be used as alternative electron acceptors. The second dominant lineage was a new species from the genus *Desulfonatronospira* (family *Desulfohalobiaceae*, order *Desulfovibrionales*). Like other members of this genus, it had the potential to reduce or disproportionate partially reduced sulfur compounds. In addition, it could also use nitrite as an alternative electron acceptor (*NrfAH*) [6].

A novel lineage of gammaproteobacterial SOB was highly abundant in the sediments of the moderately hypersaline Cock Soda Lake. It appeared as a sister group of the family *Xanthomonadaceae* in the ribosomal protein tree. This heterotrophic organism could conserve energy through aerobic respiration. It might detoxify sulfide by oxidizing it to elemental sulfur (*sqr*) with subsequent reduction or disproportionation of the polysulfides (*psrA*) chemically formed from the sulfur. It also encoded the potential for DNRA (*nrfA* and *napC*). Genes likely involved in sulfide detoxification (*sqr* and *psrA*) were found also in several other abundant MAGs of heterotrophs, including one new abundant species from the family of *Nitriliruptoraceae* (class *Nitriliruptoria,* phylum *Actinobacteria*). We found a wide variety of carbohydrate-active enzymes in these MAGs, such as cellulases (GH1 family) in addition to genes for glycolysis and TCA cycle and a chlorophyll/bacteriochlorophyll a/b synthase (*bchG* gene). The latter was also found in other *Actinobacteria* from the genus *Rubrobacter* [39]. No evidence was found for nitrile-degrading potential.

A second novel, uncultured lineage of *Gammaproteobacteria* that was highly abundant at moderate salinities branched in our ribosomal protein tree as a sister group to the family *Halothiobacillaceae*. The MAGs encoded for a versatile metabolism typical for purple non-sulfur bacteria. The MAGs contained *puf* genes, *bch* genes, genes for carotenoid biosynthesis (not shown), and a Calvin cycle for photoautotrophic growth. Alternatively, energy may be conserved through aerobic respiration, while acetate and proprionate could be taken up via an acetate permease (*actP*) and further used for acetyl-CoA biosynthesis and carbon assimilation. Since the *sqr* gene was present, but no *dsr* or *sox* genes, the organism might oxidize sulfide only to elemental sulfur. One bin contained also *nifDKH* genes suggesting putative diazotrophy, as well as a coenzyme F420 hydrogenase suggesting photoproduction of hydrogen [40].

The abundant *Euryarchaeota* organism showed a clear preference for higher salinities. We obtained one highly abundant MAG from the class *Thermoplasmata* that encoded a full-length 16S rRNA gene only distantly related (91,2% identity, *e* value 0) to that of a member of the KTK 4A group found in a hypersaline endoevaporitic microbial mat [8]. The abundant soda lake organism is likely a new genus and species. All KTK 4A-related MAGs found here encoded for similar heterotrophic, fermentative metabolisms, with the potential for anaerobic formate and CO oxidation. The KTK 4A might be also primary degraders since they encoded putative cellulases (CAZY-families GH1, GH5) and chitinases (GH18). Interestingly, half of the MAGs encoded a putative chlorophyll/bacteriochlorophyll a/b synthase (*bchG*), which is highly unusual for *Archaea*. Although little can be inferred from the presence of only one marker gene, a functional *bchG* was previously also found in *Crenarchaeota* [41]. The remaining two highly abundant *Euryarchaeota*-related MAGs belonged to a new species of *Halorubrum* (Additional file 6).

### Key genes of the Wood-Ljungdahl pathway found in novel phylogenetic groups

More than 50 MAGs were related to the family *Syntrophomonadaceae* (class *Clostridia*, phylum *Firmicutes*) based on ribosomal protein phylogeny. All 16S rRNA gene sequences found in the MAGS had 86–95% identity to sequences obtained from uncultured organisms related to the genus *Dethiobacter.* While an isolated strain of *Dethiobacter alkaliphilus* is a facultative autotroph that respires thiosulfate, elemental sulfur or polysulfides with hydrogen as an electron donor [42] or disproportionates sulfur [43], other haloalkaliphilic members of the *Syntrophomonadaceae* are reverse acetogens, oxidizing acetate in syntrophy with a hydrogenotrophic partner [44]. Two populations (different species, Additional file 6) were especially abundant in Cock Soda Lake (Fig. 4). They encoded for a full CODH/ACS complex, the key enzyme for the reductive acetyl-CoA or Wood-Ljungdahl pathway (WL) and a complete Eastern branch for CO_2_ conversion to 5-methyl-tetrahydrofolate (Additional file 9) [45, 46]. Acetogens use the WL to reduce CO_2_ to acetyl-CoA, which can be fixed into the cell or used to conserve energy via acetogenesis. Syntrophic acetate oxidizers, some sulfate reducing bacteria and aceticlastic methanogens run the WL in reverse. *Syntrophomonadaceae* sp. 2 encoded for a putative thiosulfate/polysulfide reductase as well as a phosphotransacetylase (*pta*) and an acetate kinase (*ack*) for the ATP-dependent conversion of acetate to acetyl-CoA. Although alternative pathways for the latter interconversion can exist, this second species has the complete potential for (reversed) acetogenesis.

**Fig. 6:**
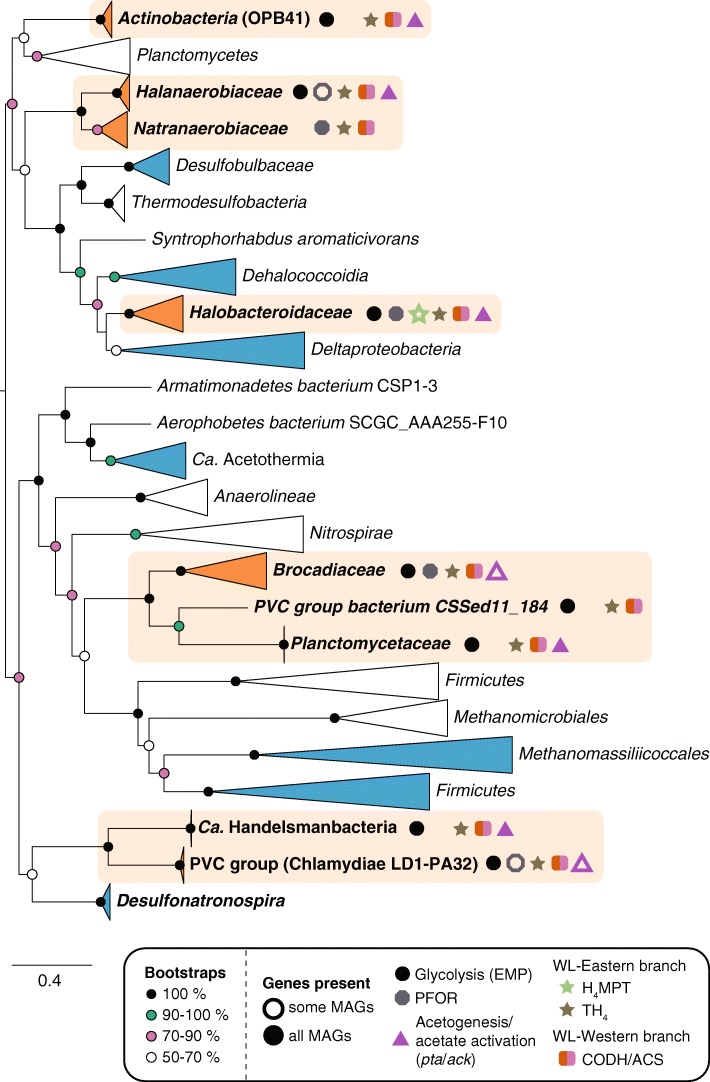
Maximum likelihood phylogeny of the bacterial-type acetyl-coA synthases (*acsB*) found in the MAGs. Only sequences ≥ 500 aa were included. Lineages for which we discovered the Wood-Ljungdahl (WL) in this study are highlighted in orange, and the presence of genes in the respective MAGs related to WL, glycolysis, pyruvate, and acetate conversion is indicated by the colored symbols (see also Additional file 9: Dataset S6). Additional lineages found in this study are marked in blue. The three was rooted according to [46]. Circles at the nodes show confidence percentage of the bootstraps analysis (100×). EMP = Embden-Meyerhof-Parnas, PFOR = pyruvate:ferredoxin oxidoreductase complex, *pta* = phosphotransacetylase gene, *ack* = acetate kinase gene, H_4_MPT = tetrahydromethanopterin-linked pathway, TH_4_ = tetrahydrofolate pathway, CODH/ACS = carbon monoxide dehydrogenase/acetyl-CoA synthase. PVC group bacterium CSSed11_184 is likely a member of the WCHB1-41 class within the *Verrucomicrobia*

Highly remarkable was the presence of a bacterial-type CODH/ACS complex and a near-complete eastern branch of the WL in a highly abundant species in Cock Soda Lake from the family *Coriobacteriaceae* (phylum *Actinobacteria*). This prompted us to scan all 871 MAGs for the presence of *acsB* encoding for the beta-subunit of the oxido-reductase module of CODH/ACS. We confirmed an encoded (near)-complete WL in several additional organisms belonging to phylogenetic groups not previously associated with this pathway [46] (Additional file 9)*.* We removed the *Coriobacteriaceae acsB* genes from the final dataset to construct a phylogenetic tree since they were < 500 aa (Fig. 6) but found seven MAGs from the OPB41 class within the *Actinobacteria* (16S rRNA gene fragment identity 94–96%). The eastern branch of WL can function independently in folate-dependent C_1_ metabolism [45], but the presence of the Western-branch in a phylum that comprises mostly aerobic isolates is very surprising. The WL in combination with the potential for acetate to acetyl-CoA interconversion (*pta*/*ack*) and a glycolysis pathway were also present in the soda lake MAGs from the phyla “Ca. Handelsmanbacteria,” “Ca. Atribacteria” (latter branched within the “Ca. Acetothermia”), and the class LD1-PA32 (*Chlamydiae*), suggesting all these uncultured organisms might be heterotrophic acetogens. However, it should be noted that a PFOR typically connecting glycolysis to the WL was only encoded in the LD1-PA32 MAGs. Moreover, from the genetic make-up alone, it cannot be excluded that acetate is activated, and the WL run in reverse for syntrophic acetate oxidation. Finally, the novel *acsB* genes from soda lake *Halanaerobiaceae*, *Natranaerobiaceae*, and *Halobacteroidaceae* (*Firmicutes*) and from *Brocadiaceae* and *Planctomycetaceae* (*Planctomycetes*) disrupt the previously proposed dichotomy between *Terrabacteria* and *Gracilicutes* bacterial groups unifying 16S rRNA and *acsB* gene phylogenies [46] and suggest a far more complex evolutionary history of the WL pathway than previously anticipated.

## Discussion

Extensive classical microbiology efforts have been already undertaken to explore the unique extremophilic microbial communities inhabiting soda lakes. These uncovered the presence of most of the functional groups participating in carbon, nitrogen, sulfur, and minor element cycling at haloalkaline conditions. The main results of this work are summarized in several recent reviews [1, 6, 47, 48]. Since most microbes, including those living in soda lakes, still evade all cultivation efforts, a very effective way to discover new microbes and assess their physiology based on their genetic repertoire is either through single cell genomics or by directly sequenced environmental DNA. This exploratory metagenomics study performed on soda lake sediments effectively overcame the existing cultivation bottleneck. First, we expanded the known diversity of CPR considerably with the first genomes of poly-extremophiles sampled from soda lake sediments. Although the presence of 16S rRNA genes from CPR in marine sediments and hypersaline microbial mats was previously shown [34], until now, CPR MAGs were mainly obtained from deep, subsurface environments [15, 26, 29, 32, 49–52], and human microbiota [30]. Despite being highly abundant here, CPR went unnoticed in previous amplicon sequencing studies. This might be due to the fact that many CPR representatives have random inserts of various length in their 16S rRNA genes or due to primer mismatches [29, 34]. This illustrates also that direct metagenomics should not only be preferred over amplicon sequencing to infer functional potential, but the former is far more effective for the discovery of novel organisms. Second, we obtained many more genomes from “traditional” bacterial phyla such as the *Planctomycetes* and *Chloroflexi*, as well as candidate phyla, for which no soda lake isolates, hence no genomes were previously obtained. Third, even within the sulfur cycle, the most active and frequently studied element cycle in soda lakes [1], we found considerable metabolic novelty. Finally, we found the Wood-Ljungdahl pathway in several novel phyla, not closely related to any known acetogens, methanogens, or sulfate-reducing bacteria [46]. The latter shows that our sequencing recovery effort has also significantly contributed to the discovery of metabolic novelty within various prokaryote phylogenetic groups.

Salinity is often considered to be the major factor shaping prokaryote community composition in diverse habitats [53, 54]. Extreme halophilic *Euryarchaeota* seem to be always the dominant group in salt-saturated hypersaline brines, both those with neutral or alkaline pH [1, 7, 37]. Here, we found that although these haloarchaea are still relatively more abundant in the sediments exposed to brines with salt-saturating conditions, the clear majority of microbes in all investigated hypersaline soda lake sediments are *Bacteria*. It could be hypothesized that the sediment is a hide-out for the extreme alkalinity and salinity governing the water column, and that sediment stratification, especially in the anoxic part, offers plenty of opportunities for niche diversification. On the other hand, it should no longer be a surprise that soda lakes are such productive and biodiverse systems. First, it has been previously elaborated that soda lake organisms are exposed to approximately half the osmotic pressure in sodium carbonate-dominated brines compared to sodium chloride-dominated brines with the same Na^+^ molarity [47]. Second, nitrogen limitation in the community can be overcome when many members contribute to the fixation of atmospheric N_2_, and various forms of organic nitrogen are efficiently recycled. The soda lakes examined in this study were also eutrophic, and sulfur compounds were abundant. Sulfide is also far less toxic at high pH as it mostly occurs in the form of bisulfide (HS^−^). Besides the evident high metabolic and taxonomic diversity of dissimilatory sulfur-cycling bacteria, a diverse heterotrophic community can be sustained comprising both generalist and very specialized carbon degraders. Less eutrophic soda lakes might not suffer from carbon limitation either, due to a presence of high-bicarbonate concentrations. These effectively eliminate the inorganic carbon limitation for primary producers who are highly active in soda lakes, especially *Cyanobacteria* [55, 56]. Third, light that penetrates the surface of the sediment seems to stimulate oxygenic and anoxygenic phototrophic growth. Moreover, various heterotrophs, such as the rhodopsin-containing haloarchaea and *Bacteroidetes*, have the option to tap into this unlimited energy source for example to help sustain the costly maintenance of osmotic balance. Unexpectedly, we even found the first rhodopsin encoded by a member of the CPR. Fourth, tight syntrophic relations, as proposed for CPR members and *Syntrophomonadaceae spp*., might be the solution to successful growth in an energetically challenging environment.

Since our metagenomes are snapshots in time and space, the failure to reconstruct specific MAGs gives no conclusive evidence for the absence of certain microbial-mediated element transformation in hypersaline soda lake sediments. Additionally, technical limitations of the assembly and binning of highly micro-diverse genome populations might hamper genome recovery [57]. More importantly, the abundance of a specific microbe is not necessarily correlated to the importance of its performance in an ecosystem. Many metabolic capacities are redundant, and often key transformations are reserved for a few rare organisms that might proliferate for a short time-span when specific conditions allow for it. For example, although no MAGs were recovered from chemolithoautotrophic nitrifiers [58], we did detect a *Nitrobacter*-related OTU by amplicon sequencing and assembled 16S rRNA genes from *Thaumarchaeota*, suggesting bacterial and archaeal nitrifiers are present in the surface sediments of soda lakes at very low abundance. Finally, the method of DNA isolation might impact the community composition apparent in the final metagenome sequenced. Environmental samples contain complex mixtures of different organisms, and it is impossible to find a protocol where the DNA from every single organism is extracted as efficiently without compromising the final quality of the extracted DNA. However, since we find all the important taxonomic and functional groups known from previous cultivation-dependent studies back in either our amplicon sequencing datasets or our directly sequenced metagenomes, we are confident that the community composition and the MAGs presented here are representative for the microbiomes of the soda lake sediments in the Kulunda Steppe.

## Conclusion

Years of intensive microbiological research on soda lakes seem to have paid off, since many of the described genera we could detect here have a cultured representative from soda lakes. However, as many of the abundant lineages and groups found in soda lake sediments are still uncultured, metagenomics proved to be a helpful tool to gain primary insights in the potential physiology and ecology of these poly-extremophilic prokaryotes. We reconstructed the first genomes for many of such organisms and proposed new functional roles for the most abundant ones. Future studies should provide more in depth analyses of these genomes, especially from the less abundant organisms that might perform key ecological processes, such as methanogens and nitrifiers. In addition, they should focus on gaining physiological culture-based evidence or proof for in situ activity for the abundant organisms described here. The key metabolic insights provided by this metagenomics study can lead to the design of new cultivation strategies. In general, sediment communities are far more complex than those found in the corresponding water column [53, 59] and are therefore often considered too complex for efficient metagenomic analysis. Many of the novel lineages found here may therefore have related neutrophilic lineages in marine and freshwater sediments that await discovery. We demonstrate here that, by providing sufficient sequencing depth, the “state of the art metagenomics toolbox” can effectively be used on sediments as well.

## Methods

### Site description and sample collection

The top 10 cm sediments from four hypersaline, eutrophic soda lakes located in the Kulunda Steppe (south-western Siberia, Altai, Russia) were sampled in July of 2010 and 2011. General features and exact location of the sampled soda lakes are summarized in Additional file 1: Table S1; a map of the area was published previously [5]. Cock Soda Lake (a stand-alone lake, sampled both in 2010 and 2011) and Tanatar-3 (Tanatar system) were moderately hypersaline (~ 100 g L^−1^) with sandy sediment, while Tanatar-1 and Bitter-1 (Bitter system) were salt-saturated (400 g L^−1^) with sulfide-rich sapropel sediments underlined by rock trona deposits [7, 60]. Especially, Bitter-1 harbors a very active microbial community, probably due to its high-organic and -mineral content. Surface sediments were collected by a plastic corer into sterile glass containers and transported to the laboratory in a cooler.

### DNA isolation, 16S rRNA amplicon, and metagenomic sequencing

The colloidal fraction of each sediment sample (~ 10% of 50 g) was separated from the course sandy fraction by several short (30–60 s) low-speed (1–2,000 rpm in 50 mL Falcon tubes) centrifugation steps and washed with 1–2 M NaCl solution. The pelleted colloidal sediment fraction was first subjected to 3 cycles of freezing in liquid nitrogen/thawing, then re-suspended in 0.1 M Tris (pH 8)/10 mM EDTA, and then subjected to harsh bead beating treatment. Next, the samples were incubated with lysozyme (15 mg/mL) for 2 h at 37 °C followed by a SDS (10% *w*/*v*) and proteinase K (10 μg/mL) treatment for 30 min. at 45 °C. High molecular weight DNA was isolated using phenol/chloroform extraction, quality-checked, and sequenced as described previously [7]. Direct high-throughput sequencing of the DNA was performed on an Illumina HiSeq 2000 platform to generate 150 b paired-end reads. Amplification of the V4-V6 region of prokaryote 16S rRNA genes using barcoded 926F-1392R primers, amplicon purification, quantification, and Roche (454)-sequencing was performed together in a batch with brine samples from the same sampling campaigns. Barcodes and adapter sequences were removed from de-multiplexed amplicon sequence reads and analyzed with the automated NGS analysis pipeline of the SILVA rRNA gene database project [61] (SILVAngs 1.3, database release version 128) using default parameters. The OTUs (97% identity) assigned down to the genus level were only considered when they had a relative abundance ≥ 0.1% in at least one of the five datasets.

### Processing metagenomics reads, assembly, binning, and post-binning

Metagenomic raw reads were quality trimmed using Sickle [62] (version 1.33), and only reads ≥ 21 b were retained. The prokaryotic community structure at taxonomic top levels was extrapolated from ten million randomly sampled singletons from each dataset. Candidate 16S rRNA fragments > 90 b were identified [63] and compared against the SILVA SSU database 128 (blastn, min. length 90, min. identity 80%, *e* value 1e-5). To verify that the microbial community composition was indeed mostly prokaryotic, we did a more general screening of the metagenomics reads that identified also candidate 18S rRNA fragments > 90 b (see Additional file 1: Tables S4-S5). The complete trimmed read sets were assembled into contigs ≥ 1 kb with MEGAHIT [64] (v1.0.3–6-gc3983f9) using paired-end mode, *k* min = 21, *k* max = 131, *k* step = 10. Genes were predicted using Prodigal [65] (v.2.6.2) and RNAs with rna_hmm3 [66] and tRNAscan-SE [67]. Assembled 16S rRNA sequences were compared to a manually curated version from the SILVA SSU database (*e* value ≥ 1e-5). Predicted protein sequences were annotated against KEGG with GhostKOALA (genus_prokaryotes + family_eukaryotes + viruses) [68]. Marker genes for central metabolic pathways and key environmental element transformations were identified based on *K* number assignments [15, 69–71].

Contigs ≥ 2.5 kb were binned with METABAT [72] (superspecific mode) based on differential coverage values obtained by mapping all five trimmed readsets to all five contig sets with Bowtie2 [73]. The bins were subjected to post-binning (an overview of the workflow is given in Additional file 2: Figure S13). Bins were assessed with lineage-specific single copy genes using CheckM [74] and further processed with the metagenomics workflow in Anvi’o [75] (v2.3.2). Since Candidate Phyla Radiant (CPR) is not included in the CheckM reference trees and are likely to have low-genome completeness, we used an existing training file of 797 CPR genomes to identify putative CPR bins [76]. Bins with CheckM-completeness ≥ 50% (884 out of 1778) and an additional four CPR bins were further processed. Coding sequences were annotated for taxonomy against NCBI-nr (July, 2017) with USEARCH [77] (5.2.32) to verify that most hits in each bin were to prokaryotic references. Phage or viral contigs were manually removed. Genome contamination (redundancy) was estimated based on marker sets of universal single copy genes identified for *Bacteria* [30] and *Archaea* [78] as implemented in Anvi’o. Genome coverage was obtained by mapping trimmed reads with BBMap [79] v36.x (kfilter 31, subfilter 15, maxindel 80). Bins with ≥ 5% redundancy were further refined with Anvi’o using circle phylograms (guide trees tnf-cov: euclidian ward) and scanned again for CPR. Post-binning resulted in a total of 2499 metagenome-assembled genomes (MAGs), of which 871 were either medium-quality genome drafts (CheckM estimated completeness ≥ 50% and contamination ≤ 10% [80], Additional file 4) or lower quality draft genomes from CPR.

Phylogeny of the MAGs was assessed based on 16 single-copy ribosomal proteins and representative reference genomes of major prokaryote lineages across the tree of life [17]. Individual ribosomal proteins in our MAGs were identified by *K* number assignments. Only ribosomal proteins ≥ 80 aa were considered. Initial maximum-likelihood (ML) trees were constructed to determine which organisms belonged to the *Archaea*, *Bacteria*, or CPR with FastTree 2 [81] (WAG + CAT). Final separate trees for the three distant evolutionary groups were constructed in the same manner. Each ribosomal protein set was aligned separately with MAFFT [82] (v7.055b, − auto) and concatenated only if a MAG encoded at least 8 out of 16 proteins. For all trees, a 100× posterior bootstraps analysis was performed. Phylogenetic trees were visualized together with genome statistics and abundance information using iTOL [83]. We cross-checked the taxonomic assignments based on the phylogeny of the ribosomal protein cassette with the top hit contig annotations against NCBI-nr and with the reference lineage obtained with CheckM. Lastly, we manually corrected the MAGs for misplaced 16S rRNA genes. The final trees presented in the manuscript were redrawn using FigTree v1.4.3 [84].

### Detailed genome analyses

CPR MAGs were re-annotated more thoroughly: genes were predicted with Prokka [85], and functional predictions were performed by running InterProScan 5 locally on the supplied COG, CDD, TIGRFAMs, HAMAP, Pfam, and SMART databases [86]. BLAST Koala was used for KEGG pathway predictions [68]. To find putative carbohydrate-active enzymes in all final MAGs, we used the web-resource dbCAN [87] to annotate all predicted proteins ≥ 80 aa against CAZy [88].

To identify the top ten abundant MAGs from each respective dataset, ten million randomly sampled singletons were mapped onto each MAG with a cut-off of 95% identity in minimum of 50 bases. Coverage values were additionally normalized for genome size and expressed as reads per kilobase of sequence per gigabase of mapped reads (RPKG) [89]. A positive score (from 871 to 1) was assigned to each MAG according to the ranking of the summed RPKG of MAGs in the high-salinity datasets (B1Sed10 and T1Sed) and a negative score according to the ranking of the summed RPKGs in the moderate salinity datasets (CSSed10, CSSed11, T3Sed10). Both scores were summed to get a “salinity preference score” with MAGs recruiting preferably from high salinity datasets on the positive end, moderate salinity datasets in the negative end, and those without preference in the middle.

We determined species delineation for the most abundant MAGs and their closest reference genomes (NCBI-nr) by Average Nucleotide Identity (ANI) and conserved DNA-matrices, as follows [90]: ANI ≥ 95%, conDNA ≥ 69% = same species, ANI ≥ 95%, condDNA < 69% = might be same species, ANI < 95%, condDNA < 69% = different species. Single gene trees based on maximum likelihood were constructed with untrimmed alignments (MAFFT, L-INS-i model) and FastTree 2 (WAG + CAT, increased accuracy, -spr4 -mlacc 2 -slownni) using 100× bootstraps. References were pulled from eggNOG (v4.5.1) [91] and supplemented with sequences from NCBI-nr or refined according to [7, 33, 46, 92–94]. The curated MAGs were scanned for the presence of rhodopsin sequences with the hmmsearch software [95] and a profile hidden Markov model (HMM) of the bacteriorhodopsin-like protein family (Pfam accession number PF01036). The identified sequences with significant similarity were aligned together with a curated database composed of a collection of type-1 rhodopsins, using MAFFT (L-INS-i accuracy model) [82]. This protein alignment was further utilized to construct a maximum likelihood tree with 100× bootstrap with FastTree 2 [81]. All other genes were identified using the KEGG annotation.

## Additional files

Additional file 1: **Table S1.** General features of the four sampled soda lakes at time of sampling. **Table S2.** SILVA classification of the 16S rRNA gene sequences found in all ≥1 kb contigs of five soda sediment metagenomic datasets. **Table S3.** Enzymes involved in lipopolysaccharide biosynthesis found among different members of the CPR. **Table S4.** Sub-kingdom classification of candidate SSU rRNA gene fragments found in subsamples of 10 million random forward reads from the five soda sediment metagenomes. **Table S5.** Top-level taxonomic classification of the 18S rRNA gene fragments found in subsamples of 10 million random forward reads from the five soda sediment metagenomes. **Table S6.** Description of the metagenomic datasets, NCBI Sequence Read Archive (SRA) accession numbers and general statistics of the assembled contigs. (PDF 740 kb)

Additional file 2: **Figure S1.** Taxonomic fingerprints determined by 16S rRNA gene amplicon sequencing. **Figure S2.** Genome statistics of the 871 MAGs. **Figure S3.** Phylogeny of MAGs belonging to “*Candidatus* Aenigmarchaeota” and “Ca. Nanohaloarchaeota”. **Figure S4.** Phylogeny of MAGs related to “*Candidatus* Acetothermia”, candidate division WS1 and “*Candidatus* Lindowbacteria”. **Figure S5.** Phylogeny of MAGs related to candidate division KSB3 and “*Candidatus* Schekmanbacteria”. **Figure S6.** Multiple sequence alignment of the V-type ATPase subunits K. **Figure S7.** Multiple sequence alignment of the F-type ATPase subunits c. **Figure S8.** Maximum likelihood tree of the large subunits of RuBisCo and RubisCo-like proteins. **Figure S9.** Maximum likelihood tree of the putative rhodopsins. **Figure S10.** Predicted isoelectric points (pI) profiles of all MAGs from CPR members. **Figure S11.** Predicted isoelectric points profiles for members of the “Ca. Nealsonbacteria” and “Ca. Vogelbacteria”. **Figure S12.** Multiple sequence alignment of the dissimilatory cytochrome c nitrite reductases (*nrfA/TvNiR*, K03385). **Figure S13.** Overview of the post-binning workflow used for genome recovery. (PDF 6548 kb)

Additional file 3: **Dataset S1.** Relative abundance of the OTUs assigned to the genus-level within the Archaea, Bacteria and organelles from Eukaryota detected by 16S rRNA gene amplicon sequencing. The OTUs with less than 0.1% abundance accross all five datasets are not shown. The names of highly abundant genera (≥1% in at least one of the datasets) are shown in bold. (XLSX 24 kb)

Additional file 4: **Dataset S2.** Organism names, statistics and general description incl. Completeness and contamination estimates, phylogeny and DDBJ/EMBL/Genbank accession numbers of the metagenome assembled genomes (MAGs) described in this paper. All submitted versions described in this paper are version XXXX01000000. **Size** = recovered genome size**,** Completeness **(Compl1)**, contamination **(Cont)**, strain heterogenity **(Str het) and Taxon CheckM** were inferred from lineage-specific marker sets and a reference tree build with CheckM [74]. Additional completeness **(compl2)** and redundancy **(red)** estimates were inferred based on the presence of universal single copy genes for *Bacteria* and *Archaea* [75]. Decision and confidence intervals from the Candidate Phyla Radiation (CPR) scan [75] are given, as well as the taxonomy of the besthit in SILVA when 16S rRNA genes were present. **Phylum/class 16 ribosomal proteins** is the taxonomy derived from our ribosomal protein trees (see main text: Figs. 2 and 3). **OTU** gives the inferred link of a population genome with our 16S rRNA gene amplicon dataset (Additional file 3). (XLSX 253 kb)

Additional file 5: **Dataset S3.** Estimated abundance and derived salinity preference from each MAG in each metagenomic dataset expressed as Reads per Kilobase of MAG per Gigabase of mapped reads (RPKG) and “salinity preference score” (see Methods section), basis for Fig. 4. (XLSX 143 kb)

Additional file 6: **Dataset S4.** Average Nucleotide Identity (ANI) and conserved DNA (condna) matrices to determine species delineation between the most abundant MAGs shown in Fig. 4, closely related (less abundant) MAGs and NCBI reference genomes. Decision matrix shows: 1 = same species, − 1 = might be same species, 0 = different species (see Methods section). (XLSX 1161 kb)

Additional file 7: **Dataset S5. Sheet 1** Presence and absence of marker genes and putative carbohydrate-active enzymes in the MAGs to infer putative roles in C, N and S element cycles based on K-number assignments and CAZy annotations. **Sheet 2** Summary basis for Fig. 4. (XLSX 41 kb)

Additional file 8: **Information S1.** More detailed description of the main metabolisms encoded by *Thioalkalivibrio*-related MAGs. **Information S2** More detailed description of the main metabolisms encoded by *Deltaproteobacterial*-related MAGs. (PDF 219 kb)

Additional file 9: **Dataset 6. Sheet 1** shows the MAGs positive for the marker gene *acsB* (K14138) encoding an acetyl-CoA synthase (ACS). The basis for Fig. 6, namely presence and absence of key genes involved in the Wood-Ljungdahly pathway, acetogenesis, methanogenesis, glycolysis and pyruvate to CO_2_ conversion is shown for each MAG. **Sheet 2** shows the MAGs positive for the marker gene *cdhC* (K00193) encoding for the beta subunit of an acetyl-CoA decarboxylase synthase complex. While *acsB* and *cdhC* correspond roughly to the *Bacterial*-type and *Archaeal*-type (methanogens) enzymes with the same function, we found few discrepancies between marker gene and genome phylogeny within the *Methanomassiliicoccaceae* and *Chloroflexi*. (XLSX 52 kb)
