# Supplementary material for: A metagenomics roadmap to the uncultured genome diversity in hypersaline soda lake sediments
Source: Microbiome. 2018 Sep 19;6:168. doi: 10.1186/s40168-018-0548-7 (PMC6146748; doi:10.1186/s40168-018-0548-7)
Supplement: Supplementary file 1 — Table S1. General features of the four sampled soda lakes at time of sampling. Table S2. SILVA classification of the 16S rRNA gene sequences found in all ≥1 kb contigs of five soda sediment metagenomic datasets. Table S3. Enzymes involved in lipopolysaccharide biosynthesis found among different members of the CPR. Table S4. Sub-kingdom classification of candidate SSU rRNA gene fragments found in subsamples of 10 million random forward reads from the five soda sediment metagenomes. Table S5. Top-level taxonomic classification of the 18S rRNA gene fragments found in subsamples of 10 million random forward reads from the five soda sediment metagenomes. Table S6. Description of the metagenomic datasets, NCBI Sequence Read Archive (SRA) accession numbers and general statistics of the assembled contigs. (PDF 740 kb) [file 40168_2018_548_MOESM1_ESM.pdf]

Table S1. General features of the four sampled soda lakes at time of sampling. Alk. = alkalinity.

**Lake**	**Cock Soda**		**Tanatar-3**	**Tanatar-1**	**Bitter-1**
**Location**	52.11N 79.17E		51.66N 79.79E	51.67N 79.81E	51.67N 79.90E
**Sediment type**	Black sand		Black sand	Black silt	Black silt
**Sampling year**	2010	2011	2010	2010	2010
**Dataset**	**CSSed10**	**CSSed11**	**T3Sed10**	**T1Sed10**	**B1Sed10**
**Salinity (g L^{-1})**	70	100	110	400	400
**pH**	10.1	10.2	10.0	9.9	10.2
**CO_3^{2-} alk. (M)**	0.34	0.96	0.50	1.20	2.00
**CO_3^{2-}/HCO_3^- alk.(M)**	0.68	1.11	1.00	2.40	4.40

Supplementary Table S2. Silva classification of the 16S rRNA gene sequences found on all ≥1kb contigs of five soda sediment metagenomic datasets. Besthits were obtained with Blastn, minimum alignment length 90 bases, minimum identity 70%.

	**% of hits**				
**Category**	**CSSed10**	**CSSed11**	**T3Sed10**	**T1Sed10**	**B1Sed10**
Archaea/Euryarchaeota	3.7	2.6	0.6	15.0	8.7
Archaea/Thaumarchaeota	1.5	2.6	2.5	2.4	4.2
Bacteria/Actinobacteria	3.4	4.4	2.9	4.5	4.5
Bacteria/Bacteroidetes	11.0	8.3	12.6	6.9	5.9
Bacteria/Caldiserica	0.2	0.2	0.2	0.0	0.0
Bacteria/Chlamydiae	0.4	0.4	0.6	0.0	1.1
Bacteria/Chloroflexi	3.2	7.2	4.0	0.8	3.5
Bacteria/Cyanobacteria	1.9	2.2	2.7	3.7	5.6
Bacteria/Deinococcus-Thermus	0.6	0.7	0.2	0.4	0.4
Bacteria/Elusimicrobia	0.6	0.7	0.4	0.8	0.7
Bacteria/Fibrobacteres	0.4	0.4	0.2	0.0	0.7
Bacteria/Firmicutes	14.4	15.4	13.4	28.5	25.8
Bacteria/Gemmatimonadetes	1.9	1.3	0.8	0.4	1.1
Bacteria/Lentisphaerae	0.6	0.7	1.1	0.8	1.4
Bacteria/Planctomycetes	6.0	6.1	5.2	2.4	6.3
Bacteria/Alphaproteobacteria	2.6	5.0	5.7	1.6	2.1
Bacteria/Betaproteobacteria	0.0	0.2	0.2	0.0	0.0
Bacteria/Deltaproteobacteria	5.4	5.4	5.4	2.4	2.1
Bacteria/Epsilonproteobacteria	0.6	0.4	0.0	0.0	0.0
Bacteria/Gammaproteobacteria	5.6	5.7	5.0	3.7	3.5
Bacteria/Spirochaetae	3.0	3.3	2.1	2.4	2.4
Bacteria/TA06	0.2	0.4	0.4	0.0	0.0
Bacteria/Tenericutes	2.6	0.7	1.9	4.1	1.4
Bacteria/Thermotogae	0.2	0.2	0.2	0.0	0.4
Bacteria/Verrucomicrobia	4.1	5.4	11.9	0.0	1.4
Unclassified	26.3	20.2	19.7	19.1	17.1
**Total # hits**	**536**	**460**	**478**	**246**	**287**

Supplementary Table S3. Enzymes involved in lipopolysaccharide biosynthesis found amongst different members of the CPR. Encoded genes *lpxH*: UDP-2,3-diacylglucosamine hydrolase (K03269), *kdtA*: 3-deoxy-D-manno-octulosonic-acid transferase (K02527), *rfaG*: UDP-glucose:(heptosyl)LPS alpha-1,3-glucosyltransferase (K02844), *rfaB*: UDP-D-galactose:(glucosyl)LPS alpha-1,6-D-galactosyltransferase (K02840), *rfaI*: UDP-glucose:(glucosyl)LPS alpha-1,3-glucosyltransferase (K03275), *rfaJ*: UDP-glucose:(galactosyl)LPS alpha-1,2-glucosyltransferase (K03279), *rfaL*: O-antigen ligase (K02847).

	**Lipid A**	**Core region**					**O-antigen**
**gene**	*lpxH*	*kdtA*	*rfaG*	*rfaB*	*rfaI*	*rfaJ*	*rfaL*
Nealsonbacteria					x	x	x
Vogelbacteria							x
Zambryskibacteria		x					x
CPR 5	x						x
Peregrinibacteria			x				x
Staskawiczbacteria			x				
Saccharibacteria			x				
Katanobacteria			x				
Dojkabacteria				x			
Falkowbacteria							x
Kaiserbacteria							x

Supplementary Table S4. Sub-kindom classification of candidate SSU rRNA gene fragments found in subsamples of 10 million random forward reads from the five soda sediment metagenomes. # rRNA frags = number of rRNA gene fragments found.

	**CSSed10**	**CSSed11**	**T3Sed10**	**T1Sed10**	**B1Sed10**
# rRNA frags >90b	9976	10612	8431	4624	9828
# rRNA frags >90b/≥70%id	5002	5334	4252	2376	4973
# 16S rRNA ~ *Bacteria*	4733	5175	4152	1717	4354
# 16S rRNA ~ *Archaea*	241	103	27	531	501
# 18S rRNA ~ *Eukarya*	28	56	73	128	118
**% *Bacteria***	**94.6**	**97.0**	**97.6**	**72.3**	**87.6**
**% *Archaea***	**4.8**	**1.9**	**0.6**	**22.3**	**10.1**
**% *Eukarya***	**0.6**	**1.0**	**1.7**	**5.4**	**2.4**

Supplementary Table S5. Top-level taxonomic classification of the 18S rRNA gene fragments found in subsamples of 10 million random forward reads from the five soda sediment metagenomes. The reads were compared against the Silva SSU database 132 (blastn, min. length 90, min. identity 70%, e-value 1e-5) and absolute read counts are shown.

	CSSed10	CSSed11	T3Sed10	T1Sed10	B1Sed10
Eukaryota/Amoebozoa	0	1	0	0	0
**Eukaryota/Archaeplastida**	**3**	**8**	**9**	**55**	**77**
Eukaryota/Excavata	0	2	1	0	0
Eukaryota/Incertae Sedis/Apusomonadidae	0	0	1	0	0
Eukaryota/Opisthokonta/Aphelidea	0	0	1	0	0
Eukaryota/Opisthokonta/Holozoa/Choanoflagellid a	0	0	2	0	0
Eukaryota/Opisthokonta/Holozoa/Ichthyosporea	0	0	0	1	0
**Eukaryota/Opisthokonta/Holozoa/Metazoa /Eumetazoa/Bilateria/Arthropoda/Crustacea**	**14**	**25**	**0**	**2**	**16**
Eukaryota/Opisthokonta/Holozoa/Metazoa /Eumetazoa/Bilateria/Arthropoda/Hexapoda	3	3	0	0	1
Eukaryota/Opisthokonta/Holozoa/Metazoa/Eumetazoa/Bilateria/Rotifera	0	0	0	0	0
Eukaryota/Opisthokonta/Holozoa/Metazoa/Eumetazoa/Bilateria/Tardigrada	0	0	0	0	2
Eukaryota/Opisthokonta/Holozoa/Metazoa/Eumetazoa/Cnidaria/Hydrozoa	0	0	1	0	0
Eukaryota/Opisthokonta/Holozoa/Metazoa/Eumetazoa/Bilateria/Rotifera	3	1	0	0	6
**Eukaryota/Opisthokonta/Nucletmycea/Fungi**	**0**	**1**	**2**	**19**	**3**
**Eukaryota/SAR/Alveolata/Ciliophora**	**3**	**1**	**0**	**48**	**4**
Eukaryota/SAR/Rhizaria/Cercozoa	0	2	3	0	1
Eukaryota/SAR/Stramenopiles/Bicosoecida	0	1	0	2	1
Eukaryota/SAR/Stramenopiles/MAST-22	0	1	0	0	0
**Eukaryota/SAR/Stramenopiles/Ochrophyta**	**1**	**8**	**49**	**0**	**5**

Supplementary Table S6. Description of the metagenomic datasets, NCBI Sequence Read Archive (SRA) accession numbers and general statistics form the assembled contigs.

**Dataset**	**CSSed10**	**CSSed11**	**T3Sed10**	**T1Sed10**	**B1Sed10**
**SRA Accession**	INGY-SRA052010	INGU-SRA052030	INGT-SRA052015	INGX-SRA052016	INGW-SRA052029
**# read pairs**	337748090	176715133	190599566	197,516,534	158975470
**Size (Gb)**	62	33.9	35.2	33.8	28.5
**# contigs >=1kb**	718182	496512	522053	213257	264614
**# contigs >=2.5kb**	178046	119339	135513	61314	74097
**Avg. contig length (bp)**	2541	2470	2704	2901	2854
**Max. contig length (bp)**	267099	216295	248848	228663	259668
**Total assembly length (bp)**	1824994602	1226438872	1411656472	618565511	755087261
**N50 (bp)**	2950	2825	3318	3757	3690
