# Supplementary material for: A metagenomics roadmap to the uncultured genome diversity in hypersaline soda lake sediments
Source: Microbiome. 2018 Sep 19;6:168. doi: 10.1186/s40168-018-0548-7 (PMC6146748; doi:10.1186/s40168-018-0548-7)
Supplement: Supplementary file 2 — Figure S1. Taxonomic fingerprints determined by 16S rRNA gene amplicon sequencing. Figure S2. Genome statistics of the 871 MAGs. Figure S3. Phylogeny of MAGs belonging to “Candidatus Aenigmarchaeota” and “Ca. Nanohaloarchaeota”. Figure S4. Phylogeny of MAGs related to “Candidatus Acetothermia”, candidate division WS1 and “Candidatus Lindowbacteria”. Figure S5. Phylogeny of MAGs related to candidate division KSB3 and “Candidatus Schekmanbacteria”. Figure S6. Multiple sequence alignment of the V-type ATPase subunits K. Figure S7. Multiple sequence alignment of the F-type ATPase subunits c. Figure S8. Maximum likelihood tree of the large subunits of RuBisCo and RubisCo-like proteins. Figure S9. Maximum likelihood tree of the putative rhodopsins. Figure S10. Predicted isoelectric points (pI) profiles of all MAGs from CPR members. Figure S11. Predicted isoelectric points profiles for members of the “Ca. Nealsonbacteria” and “Ca. Vogelbacteria”. Figure S12. Multiple sequence alignment of the dissimilatory cytochrome c nitrite reductases (nrfA/TvNiR, K03385). Figure S13. Overview of the post-binning workflow used for genome recovery. (PDF 6548 kb) [file 40168_2018_548_MOESM2_ESM.pdf]

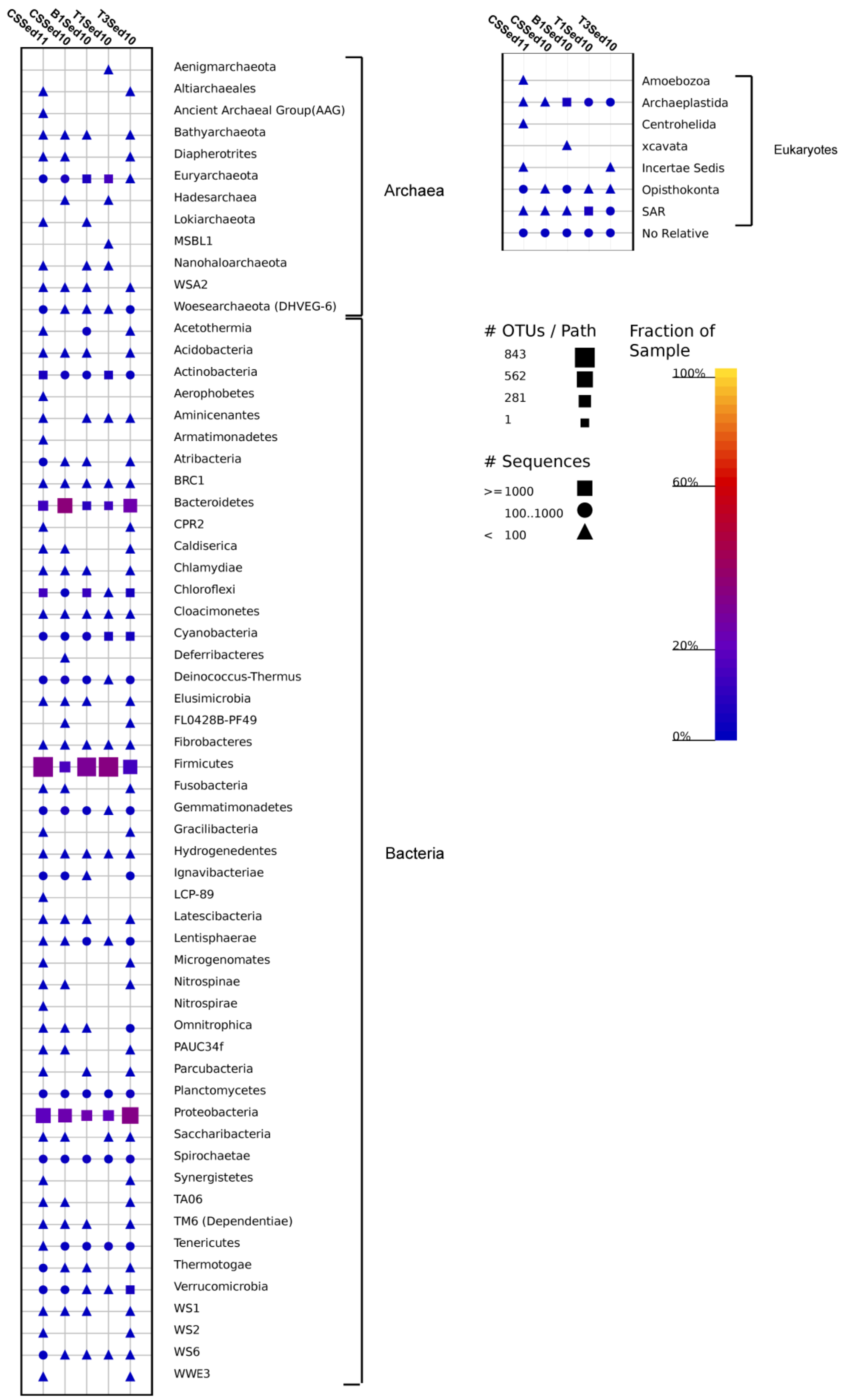

Figure S1 Taxonomic fingerprint at the phylum level of the soda sediment microbial communities determined by 16S rRNA gene amplicon sequencing. The size of the symbols is scaled to the number of sequences found per OTU, colors

indicate the fraction of a dataset that an OTU represents. Triangles, circles and squares indicate rare, moderately and highly abundant OTUs, respectively [1].

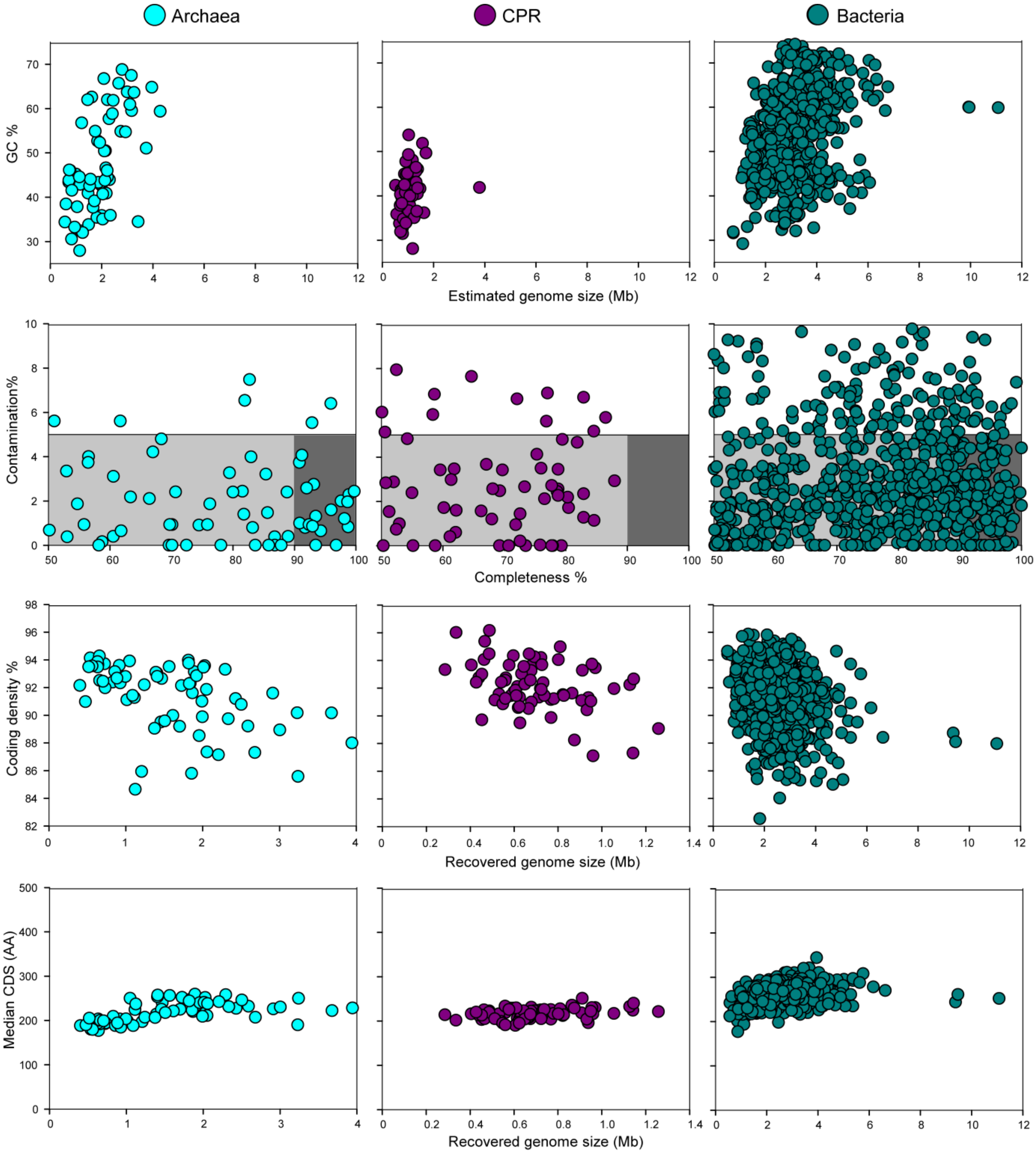

Figure S2. Summary of the genome statistics of the 871 reconstructed MAGs of this study.

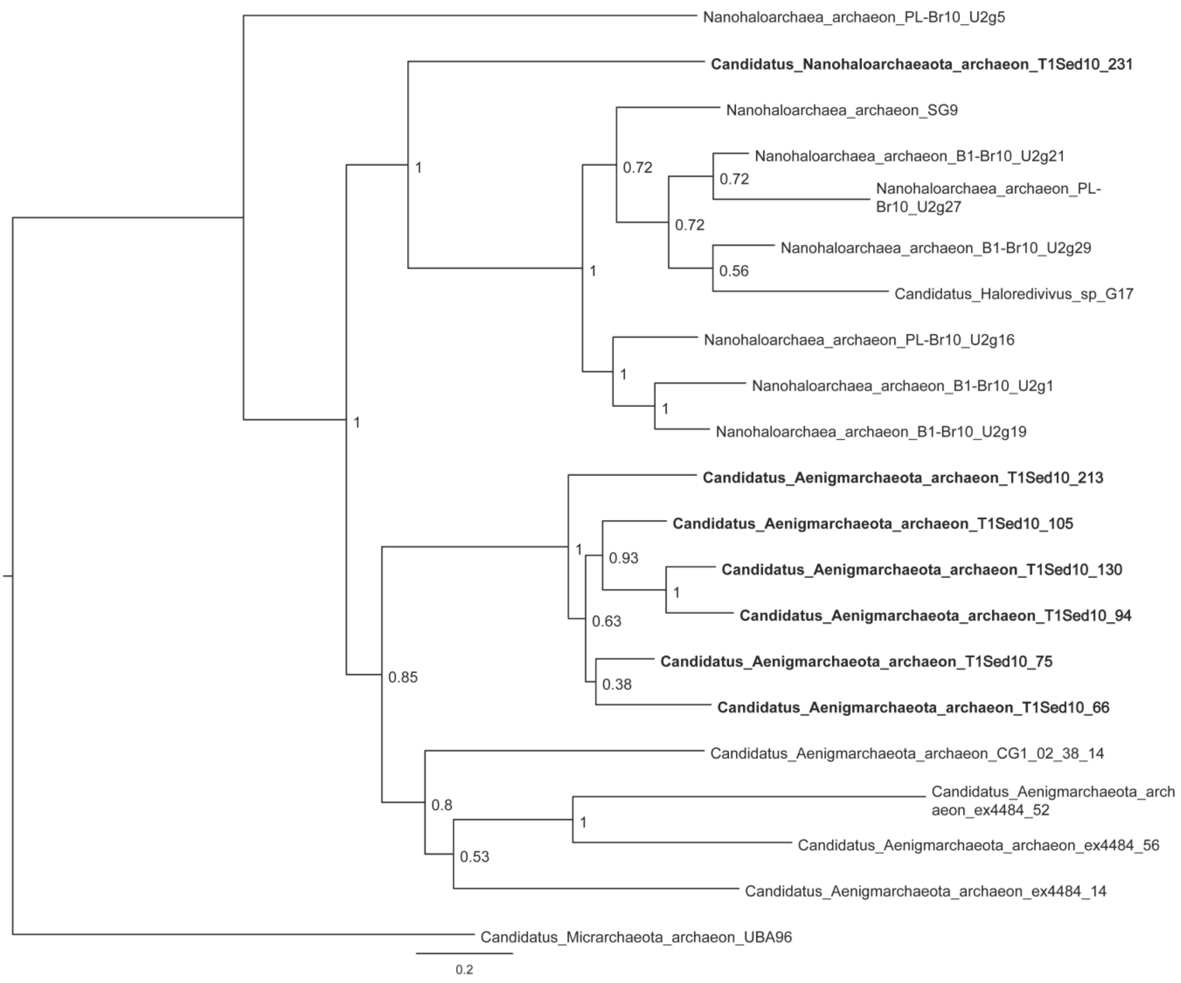

Figure S3. Maximum likelihood tree based on 20 shared COGs used to infer the phylogeny of MAGs (bold) belonging to "*Candidatus* Aenigmarchaeota" (no references included from this phylum in our ribosmomal protein tree –Figure 2: right panel) and "*Ca*. Nanohaloarchaeota" (included in ribosomal protein tree). One reference genome from the "*Ca*. Micrarchaeota" was used as an outgroup. Bootstraps values are shown at the nodes (100x).

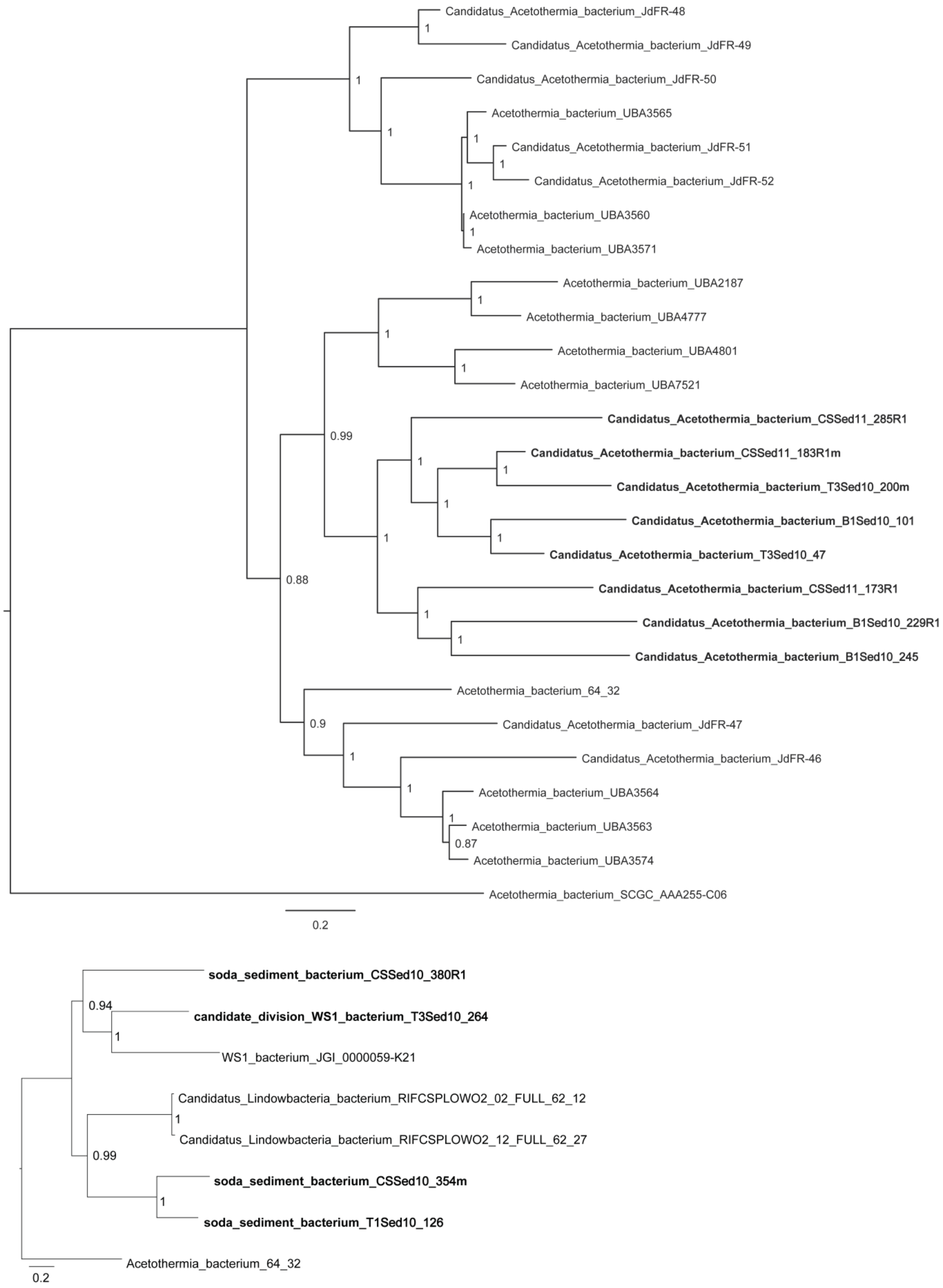

Figure S4. Maximum likelihood trees used to infer the phylogeny of MAGs (bold) related to "*Candidatus* Acetothermia" (**top panel**), candidate division WS1 and "*Candidatus* Lindowbacteria" (**bottom panel**), based on 33 and 31 shared COGs respectively. Bootstraps values are shown at the nodes (100x).

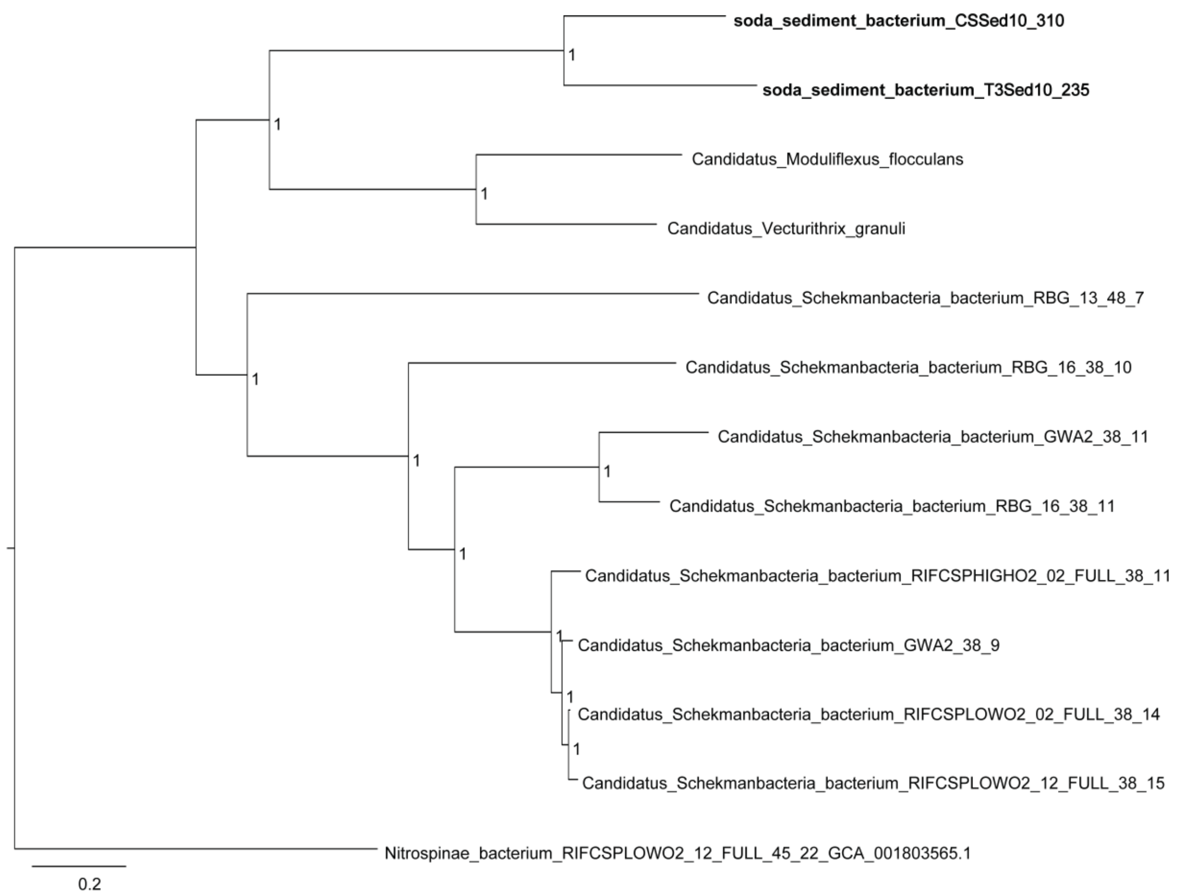

Figure S5. Maximum likelihood tree based on 133 shared COGs used to infer the phylogeny of MAGs (bold) related to candidate division KSB3 and "*Candidatus* Schekmanbacteria". One reference genome from the phylum Nitrospinae was used as an outgroup. Bootstraps values are shown at the nodes (100x).

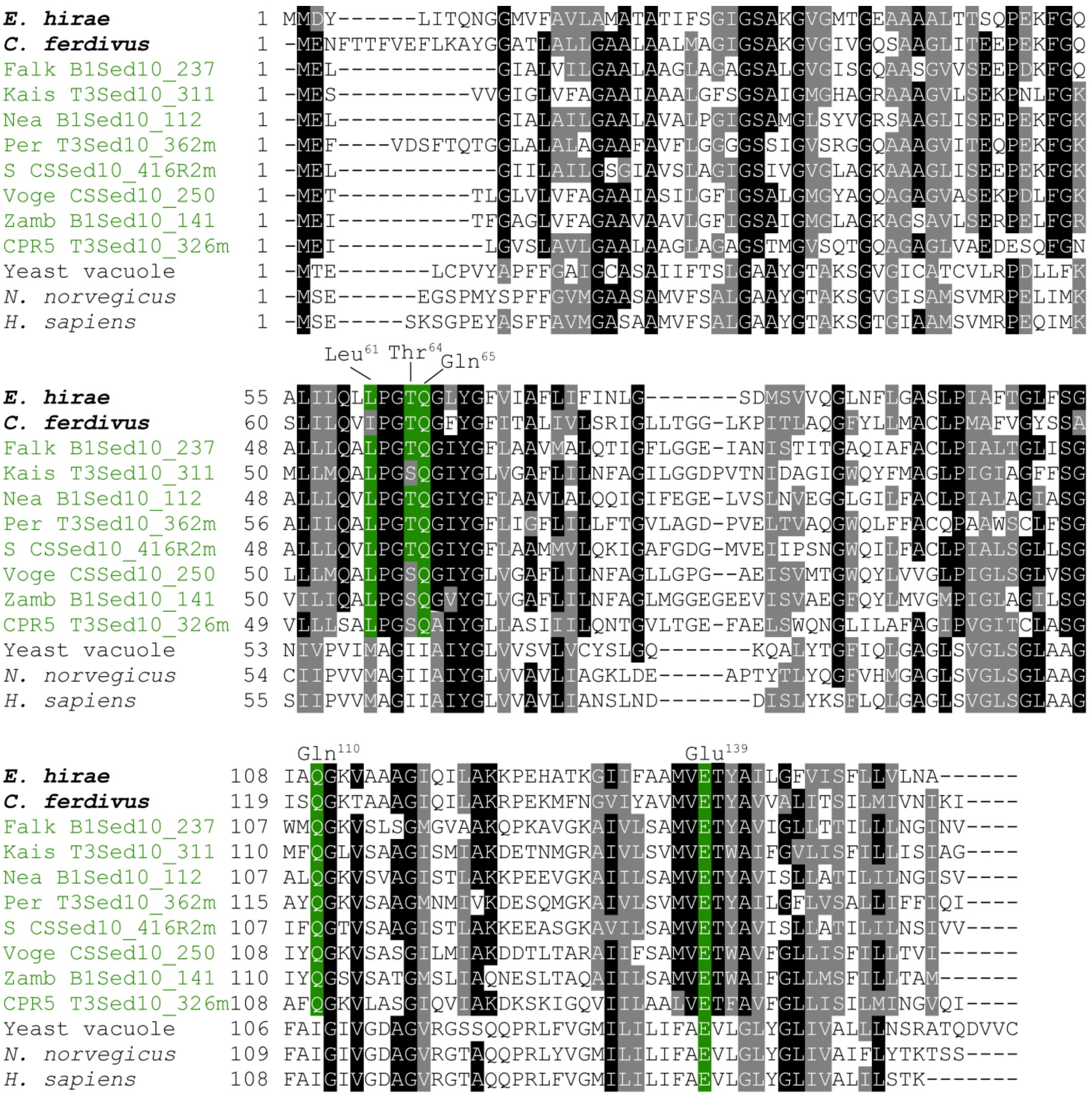

Figure S6. Multiple sequence alignment of the V-type ATPase subunit K found in all CPR MAGs, showing only a representative from each candidate phylum, and selected reference genomes [2, 3]. IDs of reference genomes and MAGs that (putatively) couple ATP hydrolysis to Na^+ translocation are shown in bold and green respectively. The conserved acidic residues forming the Na^+ binding site in *Enterococcus hirae* (Leu^{61}, Thr^{64}, Gln^{65}, Gln^{110}, Glu^{139}) are marked in green. *E.* = *Enterococcus*, *C.* = *Caloramator* (former *Clostridium*), *N.* = *Nephrops*, *H.* = *Homo*, Falk = "*Ca*. Falkowskibacteria", Kais = "*Ca*. Kaiserbacteria", Nea = "*Ca*. Nealsonbacteria", Per = "*Ca*. Peregrinibacteria", S = "*Ca*. Staskawiczbacteria", Zamb = "*Ca*. Zambryskibacteria", Voge = "*Ca.* Vogelbacteria".

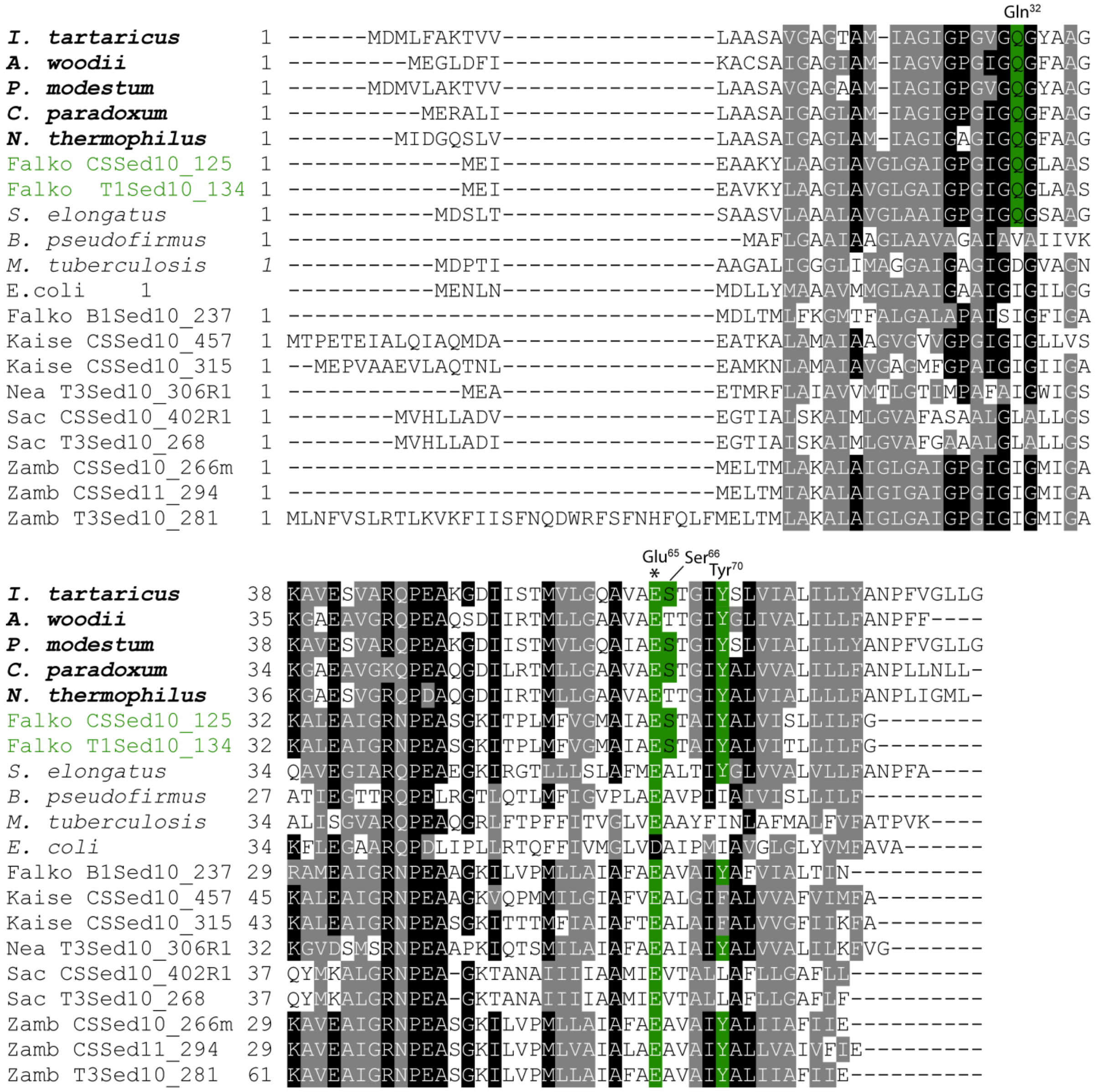

Figure S7. Multiple sequence alignment of the F-type ATPase subunits c found in all CPR MAGs and selected reference genomes [4–6]. IDs of reference genomes and MAGs that (putatively) couple ATP synthesis/hydrolysis to Na^+ translocation are shown in bold and green respectively. The conserved acidic resedue where the coupling ion (H^+/Na^+) binds is marked with an asterix. Residues that are essential for Na^+ binding in *Ilyobacter tartaricus* (Gln^{32}, Glu^{65}, Ser^{66}, Tyr^{30}) are marked in green. *A.* = *Acetobacterium*, *P.* = *Propionigenium*, *C.* = *Clostridium*, *N.* = *Natranaerobius*, Falko = "*Ca*. Falkowbacteria", Kaise = "*Ca*. Kaiserbacteria", Nea = "*Ca*. Nealsonbacteria", Sac = "*Ca*. Saccharibacteria", Zamb = "*Ca*. Zambryskibacteria".

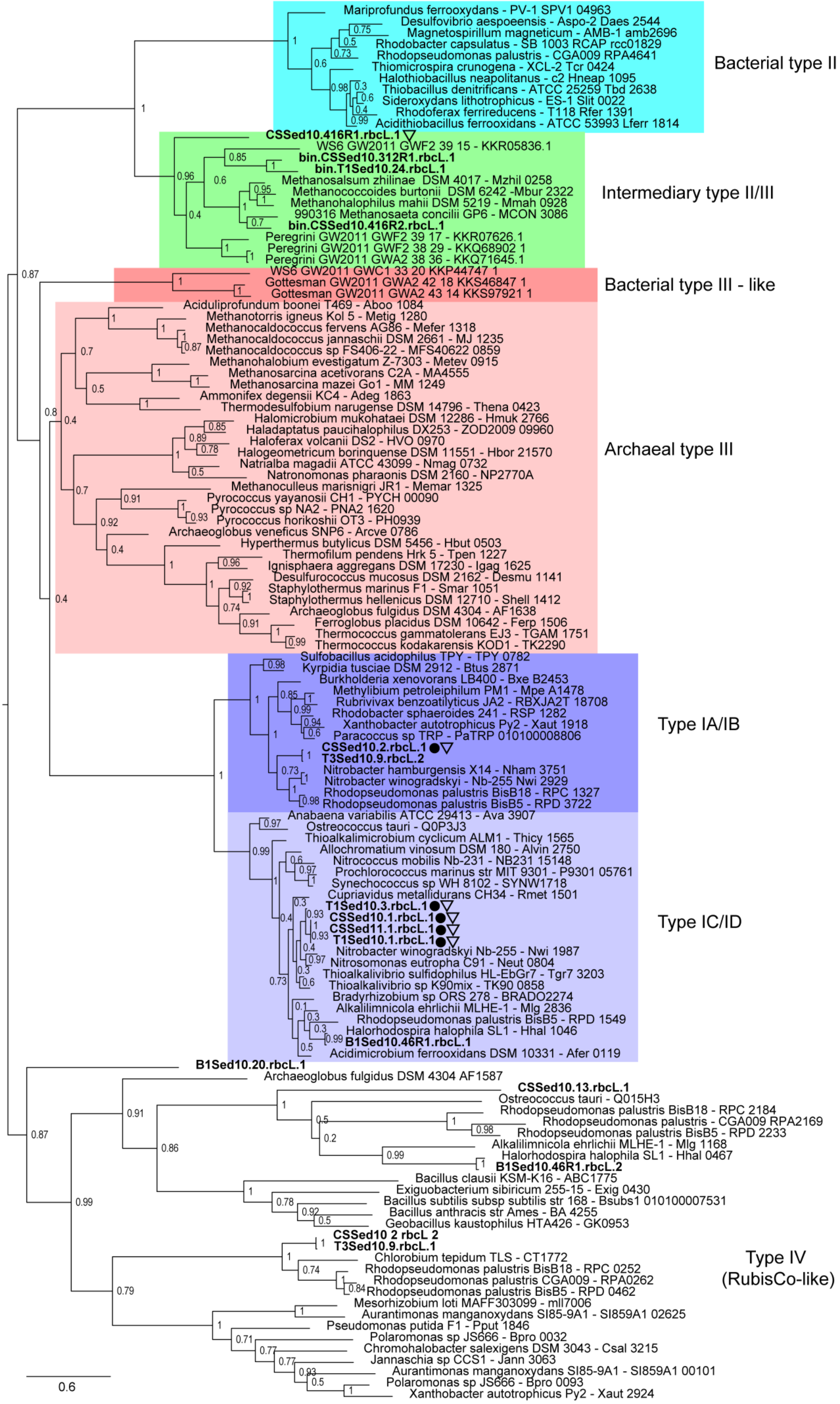

Mariprofundus ferrooxydans - PV-1 SPV1 04963
Desulfovibrio aespoeensis - Aspo-2 Daes 2544
Magnetospirillum magneticum - AMB-1 amb2696
Rhodobacter capsulatus - SB 1003 RCAP rcc01829
Rhodopseudomonas palustris - CGA009 RPA4641
Thiomicrospira crunogena - XCL-2 Tcr 0424
Halothiobacillus neapolitanus - c2 Hneap 1095
Thiobacillus denitrificans - ATCC 25259 Tbd 2638
Sideroxydans lithotrophicus - ES-1 Slit 0022
Rhodoferax ferrireducens - T118 Rfer 1391
Acidithiobacillus ferrooxidans - ATCC 53993 Lferr 1814
Bacterial type II
CSSed10.416R1.rbcL.1
WS6 GW2011 GWF2 39 15 - KKR05836.1
bin.CSSed10.312R1.rbcL.1
bin.T1Sed10.24.rbcL.1
Methanosalsum zhilinae DSM 4017 - Mzhil 0258
Methanococcoides burtonii DSM 6242 -Mbur 2322
Methanohalophilus mahii DSM 5219 - Mmah 0928
990316 Methanosaeta concilii GP6 - MCON 3086
bin.CSSed10.416R2.rbcL.1
Peregrini GW2011 GWF2 39 17 - KKR07626.1
Peregrini GW2011 GWF2 38 29 - KKQ68902 1
Peregrini GW2011 GWA2 38 36 - KKQ71645.1
Intermediary type II/III
WS6 GW2011 GWC1 33 20 KKP44747 1
Gottesman GW2011 GWA2 42 18 KKS46847 1
Gottesman GW2011 GWA2 43 14 KKS97921 1
Bacterial type III - like
Aciduliprofundum boonei T469 - Aboo 1084
Methanotorris igneus Kol 5 - Metig 1280
Methanocaldococcus fervens AG86 - Mefer 1318
Methanocaldococcus jannaschii DSM 2661 - MJ 1235
Methanocaldococcus sp FS406-22 - MFS40622 0859
Methanohalobium evestigatum Z-7303 - Metev 0915
Methanosarcina acetivorans C2A - MA4555
Methanosarcina mazei Go1 - MM 1249
Ammonifex degensii KC4 - Adeg 1863
Thermodesulfobium narugense DSM 14796 - Thena 0423
Halomicrobium mukohataei DSM 12286 - Hmuk 2766
Haladaptatus paucihalophilus DX253 - ZOD2009 09960
Haloferax volcanii DS2 - HVO 0970
Halogeometricum borinquense DSM 11551 - Hbor 21570
Natrialba magadii ATCC 43099 - Nmag 0732
Natronomonas pharaonis DSM 2160 - NP2770A
Methanoculleus marisnigri JR1 - Memar 1325
Pyrococcus yayanosii CH1 - PYCH 00090
Pyrococcus sp NA2 - PNA2 1620
Pyrococcus horikoshii OT3 - PH0939
Archaeoglobus veneficus SNP6 - Arcve 0786
Hyperthermus butylicus DSM 5456 - Hbut 0503
Thermofilum pendens Hrk 5 - Tpen 1227
Ignisphaera aggregans DSM 17230 - Igag 1625
Desulfurococcus mucosus DSM 2162 - Desmu 1141
Staphylothermus marinus F1 - Smar 1051
Staphylothermus hellenicus DSM 12710 - Shell 1412
Archaeoglobus fulgidus DSM 4304 - AF1638
Ferroglobus placidus DSM 10642 - Ferp 1506
Thermococcus gammatolerans EJ3 - TGAM 1751
Thermococcus kodakarensis KOD1 - TK2290
Archaeal type III
Sulfobacillus acidophilus TPY - TPY 0782
Kyrpidia tusciae DSM 2912 - Btus 2871
Burkholderia xenovorans LB400 - Bxe B2453
Methylibium petroleiphilum PM1 - Mpe A1478
Rubrivivax benzoatilyticus JA2 - RBXJA2T 18708
Rhodobacter sphaeroides 241 - RSP 1282
Xanthobacter autotrophicus Py2 - Xaut 1918
Paracoccus sp TRP - PaTRP 010100008806
CSSed10.2.rbcL.1
T3Sed10.9.rbcL.2
Nitrobacter hamburgensis X14 - Nham 3751
Nitrobacter winogradskyi Nb-255 Nwi 2929
Rhodopseudomonas palustris BisB18 - RPC 1327
Rhodopseudomonas palustris BisB5 - RPD 3722
Type IA/IB
Anabaena variabilis ATCC 29413 - Ava 3907
Ostreococcus tauri - Q0P3J3
Thioalkalimicrobium cyclicum ALM1 - Thicy 1565
Allochromatium vinosum DSM 180 - Alvin 2750
Nitrococcus mobilis Nb-231 - NB231 15148
Prochlorococcus marinus str MIT 9301 - P9301 05761
Synechococcus sp WH 8102 - SYNW1718
Cupriavidus metallidurans CH34 - Rmet 1501
T1Sed10.3.rbcL.1
CSSed10.1.rbcL.1
CSSed11.1.rbcL.1
T1Sed10.1.rbcL.1
Nitrobacter winogradskyi Nb-255 - Nwi 1987
Nitrosomonas eutropha C91 - Neut 0804
Thioalkalivibrio sulfidophilus HL-EbGr7 - Tgr7 3203
Thioalkalivibrio sp K90mix - TK90 0858
Bradyrhizobium sp ORS 278 - BRADO2274
Alkalilimnicola ehrlichii MLHE-1 - Mlg 2836
Rhodopseudomonas palustris BisB5 - RPD 1549
Halorhodospira halophila SL1 - Hhal 1046
B1Sed10.46R1.rbcL.1
Acidimicrobium ferrooxidans DSM 10331 - Afer 0119
Type IC/ID
B1Sed10.20.rbcL.1
Archaeoglobus fulgidus DSM 4304 AF1587
CSSed10.13.rbcL.1
Ostreococcus tauri - Q015H3
Rhodopseudomonas palustris BisB18 - RPC 2184
Rhodopseudomonas palustris - CGA009 RPA2169
Rhodopseudomonas palustris BisB5 - RPD 2233
Alkalilimnicola ehrlichii MLHE-1 - Mlg 1168
Halorhodospira halophila SL1 - Hhal 0467
B1Sed10.46R1.rbcL.2
Bacillus clausii KSM-K16 - ABC1775
Exiguobacterium sibiricum 255-15 - Exig 0430
Bacillus subtilis subsp subtilis str 168 - Bsubs1 010100007531
Bacillus anthracis str Ames - BA 4255
Geobacillus kaustophilus HTA426 - GK0953
CSSed10 2 rbcL 2
T3Sed10.9.rbcL.1
Chlorobium tepidum TLS - CT1772
Rhodopseudomonas palustris BisB18 - RPC 0252
Rhodopseudomonas palustris CGA009 - RPA0262
Rhodopseudomonas palustris BisB5 - RPD 0462
Mesorhizobium loti MAFF303099 - mll7006
Aurantimonas manganoxydans SI85-9A1 - SI859A1 02625
Pseudomonas putida F1 - Pput 1846
Polaromonas sp JS666 - Bpro 0032
Chromohalobacter salexigens DSM 3043 - Csal 3215
Jannaschia sp CCS1 - Jann 3063
Aurantimonas manganoxydans SI85-9A1 - SI859A1 00101
Polaromonas sp JS666 - Bpro 0093
Xanthobacter autotrophicus Py2 - Xaut 2924
Type IV
(RubisCo-like)
0.6

Figure S8. (Previous page) Maximum likelihood tree of the large subunit of RuBisCo (types I-III) and RuBisCo-like (type IV) proteins (*rbcL*, K01601) encoded by the most abundant MAGs (marked bold). Node values represent the bootstraps values, the scale bar gives the amino acid substitutions per site. Eggnog and genbank identifiers of the reference sequences are given next to the organisms' names. References were chosen based on previous trees constructed by [7, 8]. Phylogenetic affiliation of the abundant MAGs (see also Supplementary Dataset 2):

CSSed10.312R1 and T1Sed10.24 ~"*Ca.* Dojkabacteria";

CSSed10.416R1 ~"Ca. Peregrinibacteria";

CSSed10.416R2 ~"Ca. Staskawicsbacteria";

B1Sed10.20 ~Thermoplasmata, Euryarchaeota;

B1Sed10.46R1 ~Ectothiorhodospiraceae, Gammaproteobacteria (Halorhodospira);

CSSed10.1, bin.CSSed11. and bin.T1Sed10 ~Thioalkalivibrio sp. 1;

T1Sed10.3 ~Thioalkalivibrio sp. 2, Gammaproteobacteria;

CSSed10.2 and T3Sed10.9 ~Gammaproteobacteria ("Ca. Competibacteraceae");

CSSed10.13 ~Gammaproteobacteria (Wenzhouxiangella).

Circles and triangles indicate whether the small subunit of RubisCo (*rbcS*, K01602) or a phosphoribulokinase (*prkB*, K00855) are encoded as well.

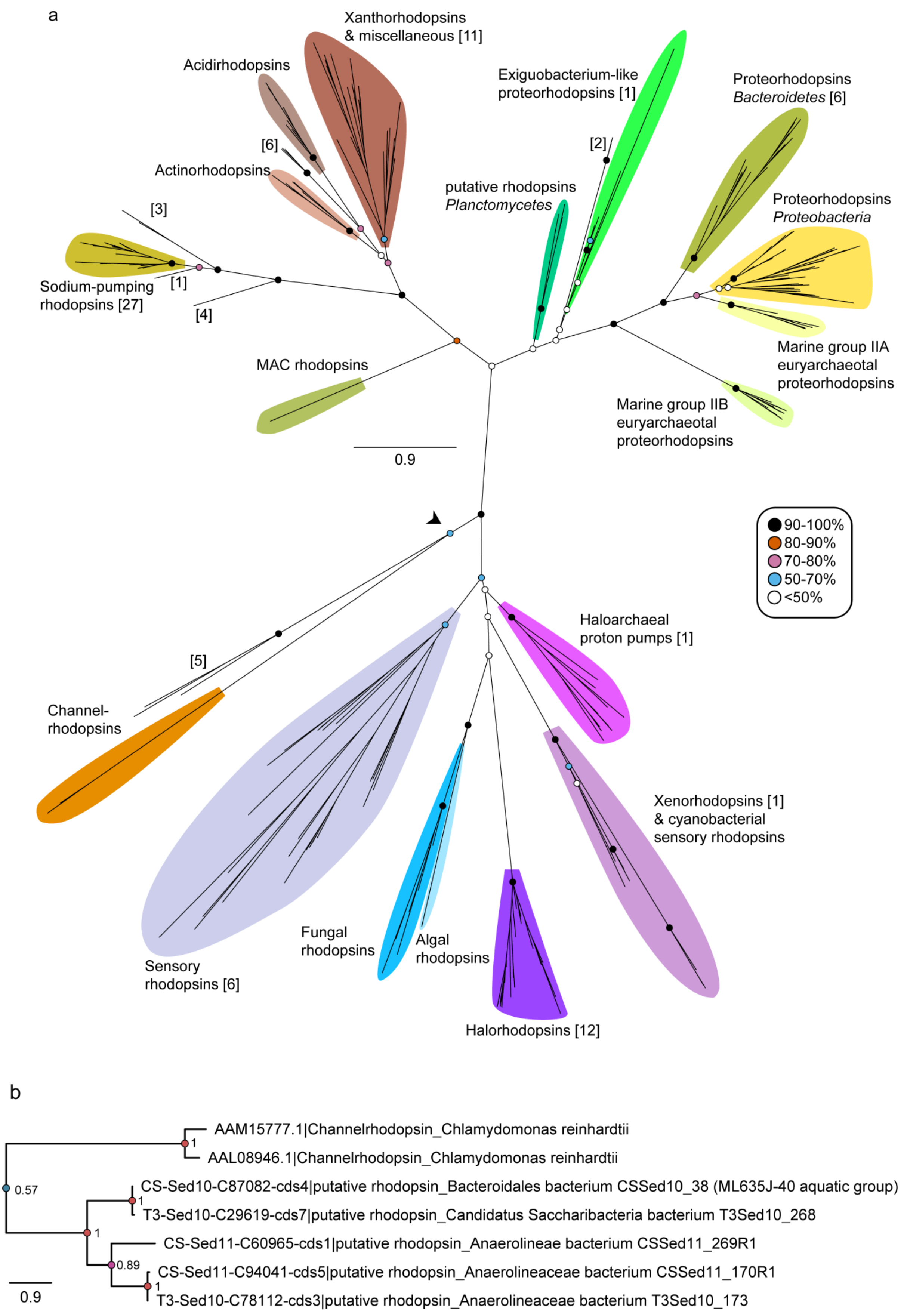
a
Xanthorhodopsins
& miscellaneous [11]
Acidirhodopsins
Exiguobacterium-like
proteorhodopsins [1]
Proteorhodopsins
Bacteroidetes [6]
[6]
[2]
Actinorhodopsins
putative rhodopsins
Planctomycetes
Proteorhodopsins
Proteobacteria
[3]
Sodium-pumping
rhodopsins [27]
[1]
[4]
Marine group IIA
euryarchaeotal
proteorhodopsins
MAC rhodopsins
Marine group IIB
euryarchaeotal
proteorhodopsins
0.9
90-100%
80-90%
70-80%
50-70%
<50%
Haloarchaeal
proton pumps [1]
[5]
Channel-
rhodopsins
Xenorhodopsins [1]
& cyanobacterial
sensory rhodopsins
Fungal
rhodopsins
Algal
rhodopsins
Sensory
rhodopsins [6]
Halorhodopsins [12]
b
AAM15777.1|Channelrhodopsin_Chlamydomonas reinhardtii
AAL08946.1|Channelrhodopsin_Chlamydomonas reinhardtii
CS-Sed10-C87082-cds4|putative rhodopsin_Bacteroidales bacterium CSSed10_38 (ML635J-40 aquatic group)
T3-Sed10-C29619-cds7|putative rhodopsin_Candidatus Saccharibacteria bacterium T3Sed10_268
CS-Sed11-C60965-cds1|putative rhodopsin_Anaerolineae bacterium CSSed11_269R1
CS-Sed11-C94041-cds5|putative rhodopsin_Anaerolineaceae bacterium CSSed11_170R1
T3-Sed10-C78112-cds3|putative rhodopsin_Anaerolineaceae bacterium T3Sed10_173
1
0.57
1
1
0.89
1
0.9

Figure S9. (Previous page) Maximum likelihood tree of the putative rhodopsins found in the recovered MAGs **a)** overview tree showing all known functional groups. Values in square brackets indicate the number of sequences found in our datasets. Black arrow indicates position of **b)** the branch with the putative channelrhodopsin found in a member of the CPR (*Candidatus* Saccharibacteria bacterium T3Sed10.268). The scale bar gives the amino substitutions per site, colored circles at selected nodes indicate the 100x bootstraps values.

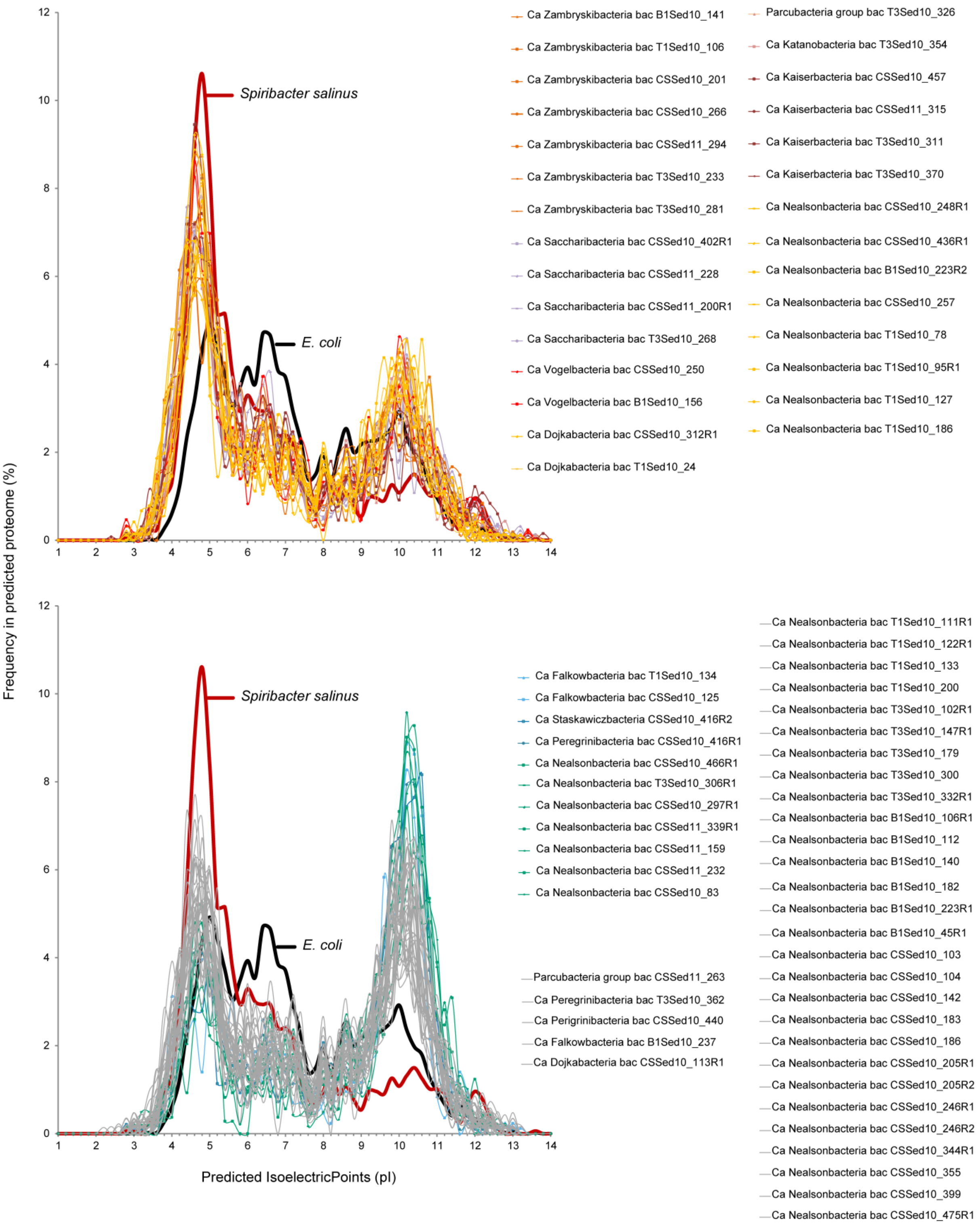

Figure S10. Predicted isoelectric points (pI) for the coding sequences in the predicted proteomes of all MAGs from CPR members. **Top panel:** MAGs with an acidic proteome. **Bottom panel**: Mags with a basic (blue colors) or bimodal (grey) distribubated proteomes. The genomes of the *Gammaproteobacteria E. coli* (neutrophile) and the halophile *S. salinus* (acidic proteome) were chosen as a reference.

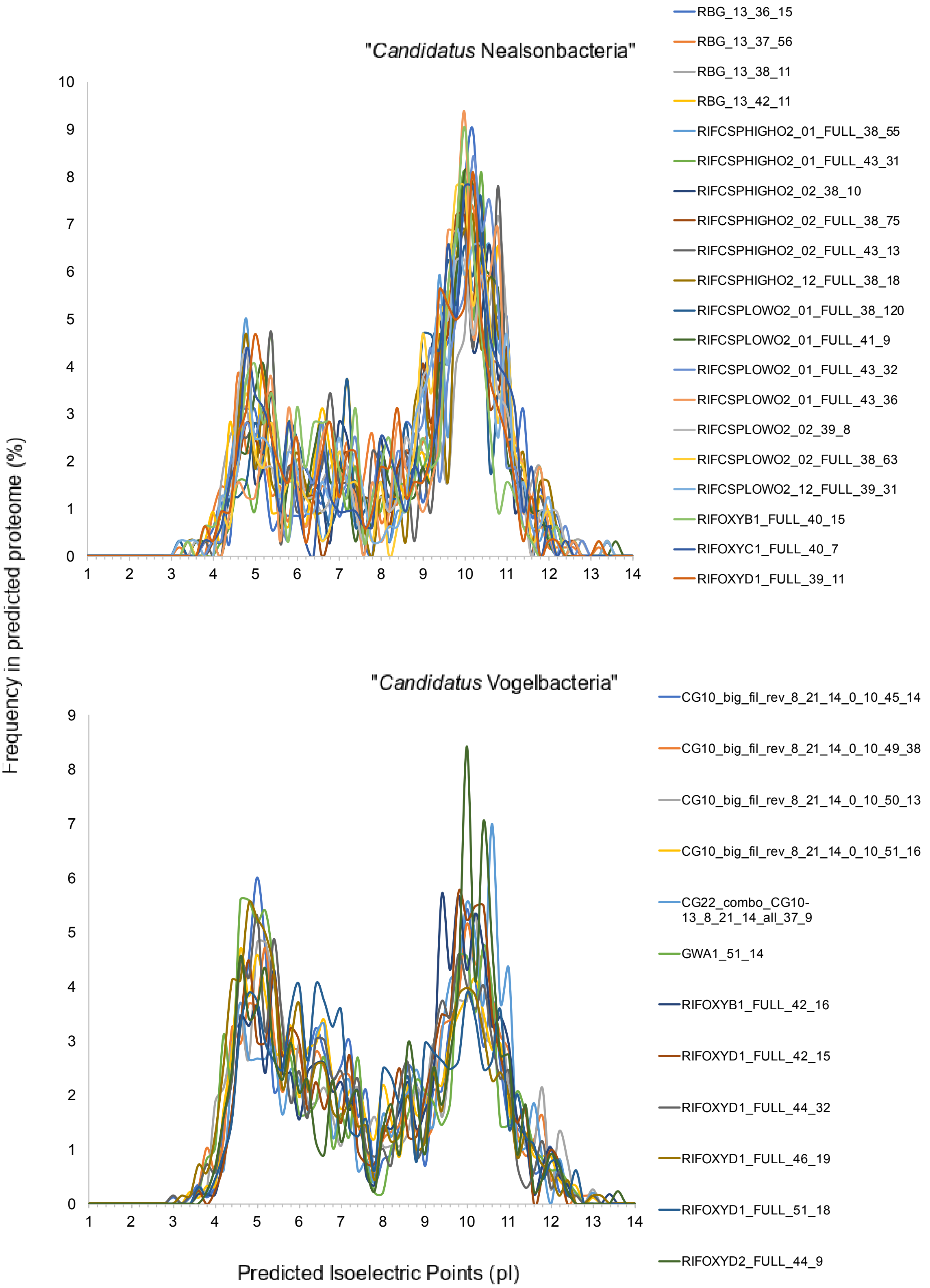

Figure S11. Predicted isoelectric points (PI) for the coding sequences in the predicted proteomes from reference genomes belonging to the "Ca. *Nealsonbacteria*" (**Top panel)** and "Ca. *Vogelbacteria*" (**Bottom panel**).

```
B1Sed10_47R1_1       1 MSDHIGVGRIGRWVAGAACLLFVTTAHAMP---GEKLKPVDALQCFDCHTQIEDMHTVGKHATVNCVHCH
B1Sed10_47R1_2       1 MRYLLSLAAGIFLA--------------------------------------------------------
B1Sed10_5_1          1 MRHIVILAASAILF--------------------------------------------------------
B1Sed10_5_2          1 MITVNNVGRMGRWVAGAACLLIMATTHAT----AENLKPVDAMQCYDCHAQIEDMHTVGKHATVNCVHCH
CSSed11_246R1_1      1 MTSSHGFGHIGRWVAGAACLLIMTTAHAMPGGGKQPLKPVDTLQCYDCHAQIEDMHAENKHATVNCVHCH
CSSed11_34_1         1 MITVNNVGRMSRWVAGAACLLIMTTTHAT----AENLKPVDAMQCYDCHAQIEDMHTVGKHATVNCVHCH
T1Sed10_3_1          1 MRHIVILAASAILF--------------------------------------------------------
T1Sed10_3_2          1 MITVNNVGRMGRWVAGAACLLIMATTHAT----AENLKPVDAMQCYDCHAQIEDMHTVGKHATVNCVHCH
Tv_nitratireducens   1 MNDLNRLGRVGRWIAGAACLFLASAAHAEP---GENLKPVDAMQCFDCHTQIEDMHTVGKHATVNCVHCH
Tv_paradoxus         1 MNDLNRLGRVGRWVAGAACLFLASAAHAGS---GDQLKPVDALQCYDCHTQIEDMHVVGKHATVNCVHCH

B1Sed10_47R1_1      68 DASAHVESASTRRMGERPVTRMDH--------EACATCHMAQY-NSFVS-------------------VR
B1Sed10_47R1_2      15 --------------GASMAVNASE-WELGEGNKAFQ-HLP-QY-QSYLK------NYDDTQMTEYGGSVP
B1Sed10_5_1         15 --------------GPVLTAGAEE-WELGEGNKAFQ-HLP-QY-QSYLK------NYDDTQMTEYGGSVP
B1Sed10_5_2         67 DASEHVETASTRRMGERPVTHTSP--------EACASCHTAQF-NSFVN-------------------VR
CSSed11_246R1_1     71 DASKHVETASTRRMGERPITHTSP--------EACASCHTAQF-NSFVS-------------------VR
CSSed11_34_1        67 DASEHVETASTRRMGERPVTRMDL--------EACATCHTAQF-NSFVH-------------------VH
T1Sed10_3_1         15 --------------GPVLTAGAEE-WELGEGNKAFQ-HLP-QY-QSYLK------NYDDTQMTEYGGSVP
T1Sed10_3_2         67 DASEHVETASTRRMGERPVTHTSP--------EACASCHTAQF-NSFVN-------------------VR
Tv_nitratireducen   68 DATEHVETASSRRMGERPVTRMDL--------EACATCHTAQF-NSFVE-------------------VR
Tv_paradoxus        68 DATEHVETASARRMGERPVTHTSP--------EACASCHTAQF-NSFAS-------------------VR

B1Sed10_47R1_1     110 HKSH--PREEKANPRSRSPKFDTLIGQHGFSFEHAEPRSHAFMLVDHFIVDRAYGGRFQYQDWTKVTDGI
B1Sed10_47R1_2      61 HRKHDDFNPLPKGYKHAQPYLKNLWLGYPFSYQYDRARGHVYALEDVLKIDRI-----------------
B1Sed10_5_1         61 HRKHDDFNPLPKGYKHAQPYLKNLWLGYPFSYEYDRARGHVYSIEDVTHIDRI-----------------
B1Sed10_5_2        109 HESH--PRVEKATPTSRSPMFDKLIAGHGFALEHAEPRSHAFMLVDHFVVDRAYGGRFQWQDWTKVSDGM
CSSed11_246R1_1    113 HESH--PRLEKANPISRSPKFDKLIGGHGFSFEHAEPRSHAFMLVDHFIVDRAYGGRFQYKNWQKVTDGM
CSSed11_34_1       109 HESE--PRVEKATPTSRSPMFDKLIAGHGFAFEHAEPRSHAFMLVDHFVVDRAYGGRFQWQDWTKVSDGM
T1Sed10_3_1         61 HRKHDDFNPLPKGYKHAQPYLKNLWLGYPFSYEYDRARGHVYSIEDVTHIDRI-----------------
T1Sed10_3_2        109 HESH--PRVEKATPTSRSPMFDKLIAGHGFALEHAEPRSHAFMLVDHFVVDRAYGGRFQWQDWTKVSDGM
Tv_nitratireduc    110 HESH--PRLEKATPTSRSPMFDKLIAGHGFAFEHAEPRSHAFMLVDHFVVDRAYGGRFQFKNWQKVTDGM
Tv_paradoxus       110 HESH--PREEKANPRSRSPKFDTLIGAHGFSLEHAEPRSHAFMLVDHFIVDRAYGGRFQYKSWQNVTDGL

B1Sed10_47R1_1     178 GAARGAWTVLKDLDPTTSDQRRFLSQTATAANPVCLNCKTQDHLLDWAYMGDEHPAAKWARTSNVVEFAR
B1Sed10_47R1_2     114 --------------------DRYSEQAALPS--TCYNCKTNTIPQLLEKHGDDFWAMNFHDFRKMHDPK-
B1Sed10_5_1        114 --------------------NRYSEQAGLPT--TCWNCKTNTMPQLLEEHGDALWSMNFHDFRKMHDPA-
B1Sed10_5_2        177 GSVQGAWTVLKDADPESSDQRRFLSQTATAANPVCLNCKTQDHILDWAYMGDEHDAAKWSRTSQVVDFAR
CSSed11_246R1_1    181 GAVRGAWTVLTDADPESSDQRRFLSQTATAANPVCLNCKTQDHILDWAYMGDPHSAAKWDRTSQVVEFAR
CSSed11_34_1       177 GSVQGAWTVLKDMDPETSDQRRFLSQTATAANPVCLNCKTQDHILDWAYMGDEHDAATWSRTSNVVEFAR
T1Sed10_3_1        114 --------------------NRYSEQAGLPT--TCWNCKTNTMPQLLEEHGDALWSMNFHDFRKMHDPA-
T1Sed10_3_2        177 GSVQGAWTVLKDADPESSDQRRFLSQTATAANPVCLNCKTQDHILDWAYMGDEHDAAKWSRTSQVVDFAR
Tv_nitratireduc    178 GAVRGAWTVLTDADPESSDQRRFLSQTATAANPVCLNCKTQDHILDWAYMGDEHEAAKWSRTSEVVEFAR
Tv_paradoxus       178 GAVRGAWTVIEDMDPTTSDQRRFLAQTATAANPVCLNCKTQDHILDWAYMGDEHDAAKWARTSKVVDFAR

B1Sed10_47R1_1     248 DLHHPLNCFMCHDPH-SAGPRVVRDGLIHAVVDQGLGTYPYDAAKSERITLTPVTFQREGQDFRKIGLLN
B1Sed10_47R1_2     161 --RHTIGCTNCHDPENQMRLTITSVPLTEAL-------------------------ERQGKDWRNAS--R
B1Sed10_5_1        161 --RHSIGCTNCHDPANQMRLAITSVPLTEAL-------------------------ERQGKDWRNAS--R
B1Sed10_5_2        247 DLHHPVNCFMCHDPH-SAEPRVVRDGLIHAVIDQGLGTYPNDPEKSAMLSMEKVTFQRGGEDFRAIGLLS
CSSed11_246R1_1    251 DLHHPVNCFMCHDPH-SAEPRVVRDGLIHAVADRELGTYPNDPAKSAMLSMEKVTFQRGGEDFRAIGLLS
CSSed11_34_1       247 DLNHPLNCFMCHDPH-SAGPRVVRDGLIHAVVEQGLGTYPQDAEKSELITMEKVTFQRGGEDFRAIGLLS
T1Sed10_3_1        161 --RHSIGCTNCHDPANQMRLAITSVPLTEAL-------------------------ERQGKDWRNAS--R
T1Sed10_3_2        247 DLHHPVNCFMCHDPH-SAEPRVVRDGLIHAVIDQGLGTYPNDPEKSAMLSMEKVTFQRGGEDFRAIGLLS
Tv_nitratireduc    248 DLNHPLNCFMCHDPH-SAGPRVVRDGLINAVVDRGLGTYPHDPVKSEQQGMTKVTFQRGREDFRAIGLLD
Tv_paradoxus       248 DLHHPVNCYMCHDPH-STEPRVVRDALIHAVVDQGLGTYPYDEAKSEHVTLTPVTFQRGGEDFRKIGLLN

B1Sed10_47R1_1     317 VADSNLMCGQCHVEYNCNPGFQQSDNAPVGMDDRRTNHFFW------ANVFDYKEAAKRID-------FF
B1Sed10_47R1_2     202 NEMRSLVCAQCHVEYYFETAEHGVAAKPH---------LPWDKGMDPEDMYRFKAD-GDPERDGFSGQFR
B1Sed10_5_1        202 DEMRSLVCAQCHVEYYFETADHGIAAKPH---------FPWDKGMNAEDIYRFKAD-GDPERDGFKGQFS
B1Sed10_5_2        316 TPDSNLMCAQCHVEYNCNPGSQLSDGARVGMDDRRANHFFW------ANVFDYREAAERID-------FF
CSSed11_246R1_1    320 TADSNLMCAQCHVEYNCNPGFQLSDGSRVGMDDRRANHFFW------ANVFDYKEAAQEID-------FF
CSSed11_34_1       316 MPDSNLMCAQCHVEYNCNPGSQLSDGARVGMDDRRANHFFW------ANVFDYKEAAERID-------FF
T1Sed10_3_1        202 DEMRSLVCAQCHVEYYFETADHGIAAKPH---------FPWDKGMNAEDIYRFKAD-GDPERDGFKGQFS
T1Sed10_3_2        316 TPDSNLMCAQCHVEYNCNPGSQLSDGARVGMDDRRANHFFW------ANVFDYREAAERID-------FF
Tv_nitratireduc    317 TADSNVMCAQCHVEYNCNPGYQLSDGSRVGMDDRRANHFFW------ANVFDYKEAAQEID-------FF
Tv_paradoxus       317 VADSNLMCGQCHVEYNCNPGFQQSDGAPVGMDDRRTNHFFW------ANVFDYAEAAKEID-------FF
```

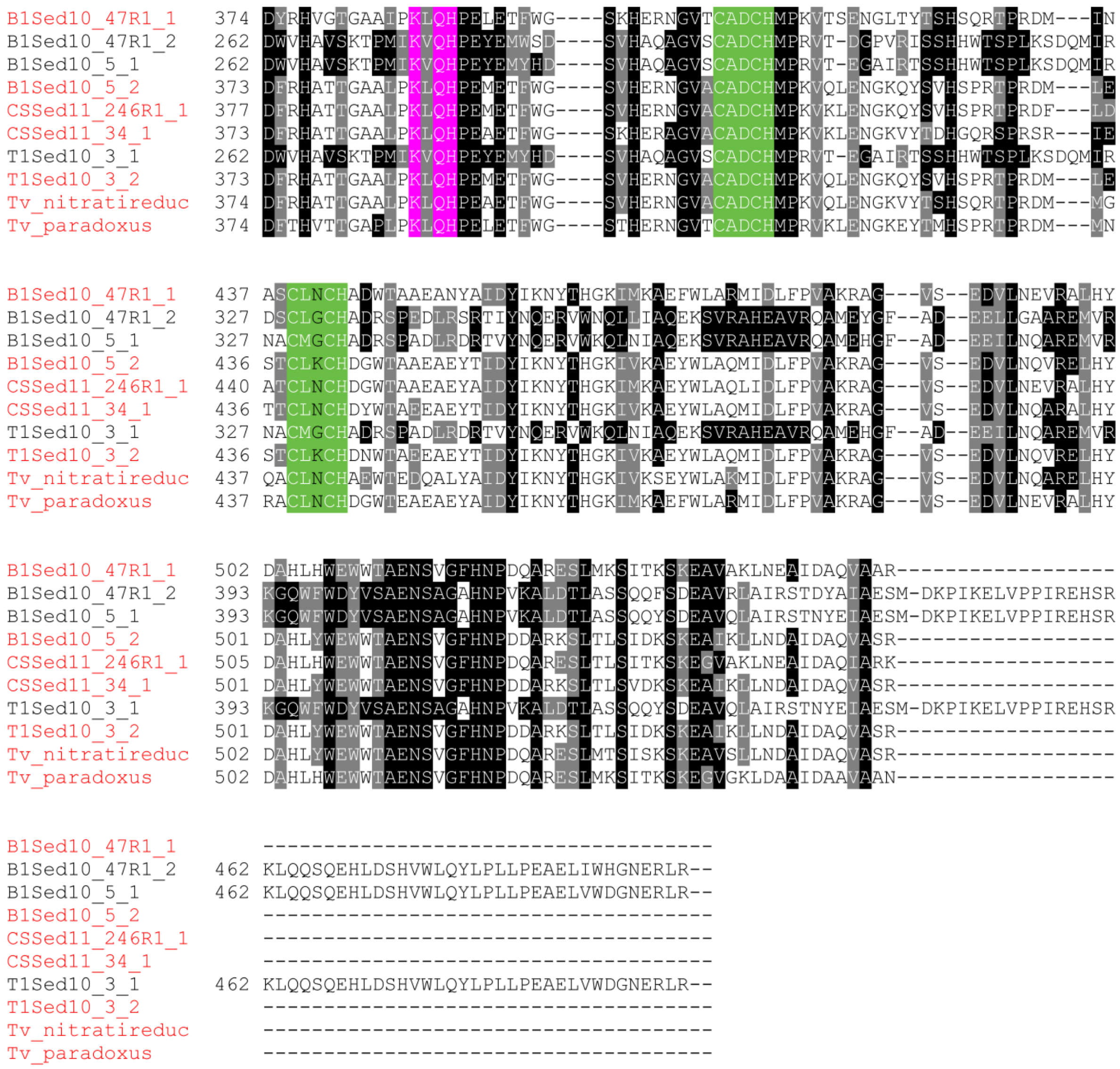

Figure S12. Multiple sequence alignment of the dissimilatory cytochrome c nitrite reductases (*nrfA/TvNiR*, K03385) encoded by the most abundant MAGs, by less abundant MAGs from related phylogenetic groups and two reference genomes. Here we show only MAGs where we found a octaheme type encoded by the *TvNiR* gene (red ID). Few genomes encoded additionally for the more common pentaheme type (*nrfA*, black ID). Grey-Black shading indicates conserved motifs (>50-100% homology). Overlaid, we highlighted in **light green** characteristic haem *c* binding motifs CXXCH (7 for the TvNir type), in **dark green** the special CXXCK motif. Locations of the active site cavities are shown in **magenta**. CSSed11_34 was a member of the family *Desulfobacteraceae*. Both B1Sed10_5 and T1Sed10_3 belonged to the abundant *Thioalkalivibrio* sp. 2 (see Figure 4 main text), B1Sed10_47R1 and CSSed11_246R1 belonged to less abundant *Thioalkalivibrio* spp.

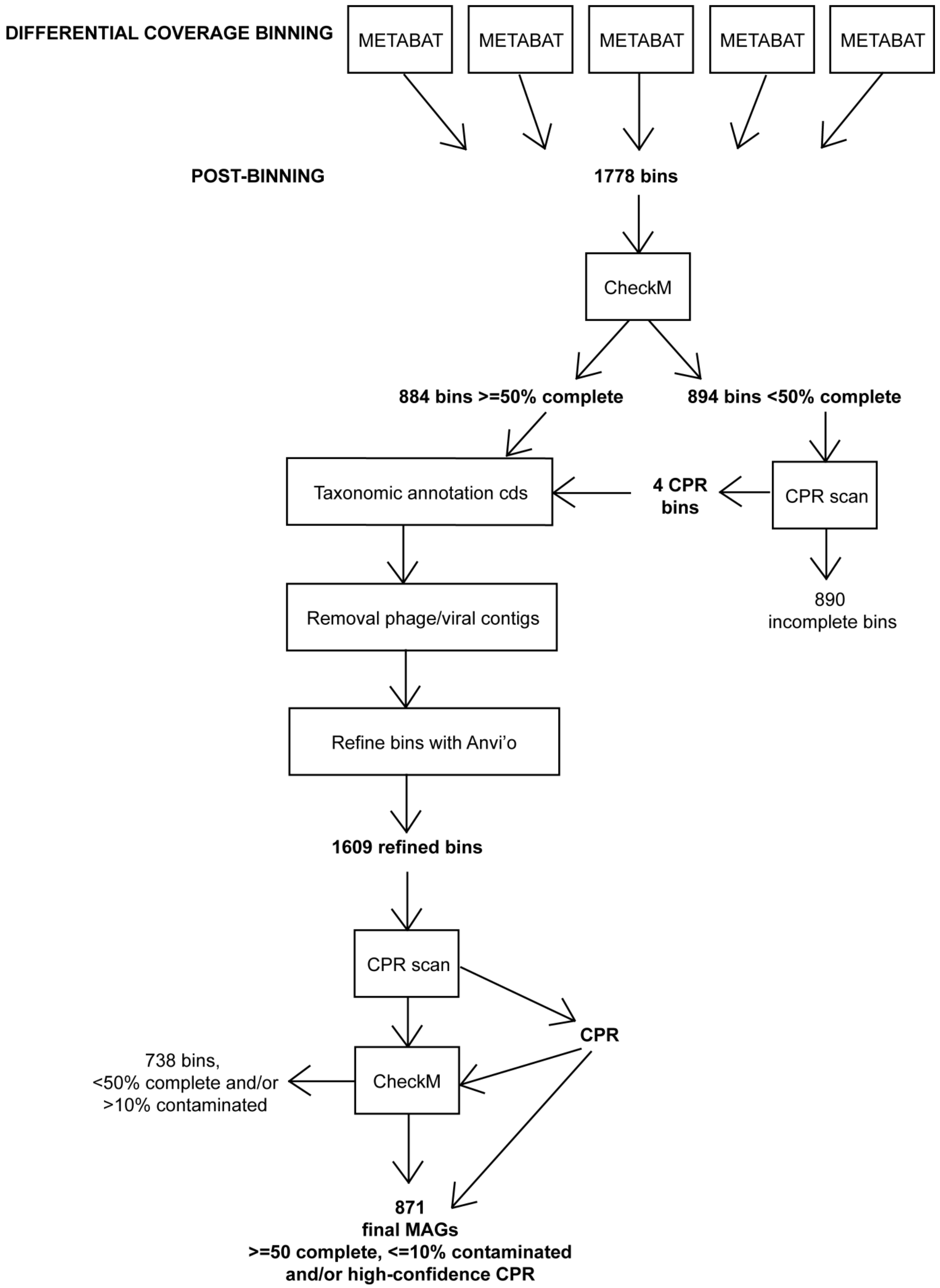

Figure S13 Overview of the post-binning workflow used for genome recovery after automated assembly and binning (METABAT) of five separate datasets.

References

1. Quast C, Pruesse E, Yilmaz P, Gerken J, Schweer T, Yarza P, et al. The SILVA ribosomal RNA gene database project: improved data processing and web-based tools. Nucleic Acids Res. 2012;41:D590–6.

2. Murata T. Structure of the rotor of the V-Type Na^+-ATPase from *Enterococcus hirae*. Science (80-). 2005;308:654–9.

3. Speelmans G, Poolman B, Abee T, Konings WN. Energy transduction in the thermophilic anaerobic bacterium *Clostridium fervidus* is exclusively coupled to sodium ions. Proc Natl Acad Sci. 1993;90: 7975-7979..

4. Meier T. Structure of the Rotor Ring of F-Type Na^+-ATPase from *Ilyobacter tartaricus*. Science. 2005;308:659–62.

5. Ferguson SA, Keis S, Cook GM. Biochemical and molecular characterization of a Na^+-translocating F_1F_o-ATPase from the thermoalkaliphilic bacterium *Clostridium paradoxum*. J Bacteriol. 2006;188:5045–54.

6. Mesbah NM, Cook GM, Wiegel J. The halophilic alkalithermophile *Natranaerobius thermophilus* adapts to multiple environmental extremes using a large repertoire of $Na^+(K^+)/H^+$ antiporters. Mol Microbiol. 2009;74:270–81.

7. Tabita FR, Hanson TE, Li H, Satagopan S, Singh J, Chan S. Function, structure, and evolution of the RuBisCO-like proteins and their RuBisCO homologs. Microbiol Mol Biol Rev. 2007;71:576–99.

8. Wrighton KC, Castelle CJ, Varaljay VA, Satagopan S, Brown CT, Wilkins MJ, et al. RuBisCO of a nucleoside pathway known from *Archaea* is found in diverse uncultivated phyla in bacteria. ISME J. 2016;10:2702–14.
