# Supplementary material for: A metagenomics roadmap to the uncultured genome diversity in hypersaline soda lake sediments
Source: Microbiome. 2018 Sep 19;6:168. doi: 10.1186/s40168-018-0548-7 (PMC6146748; doi:10.1186/s40168-018-0548-7)
Supplement: Supplementary file 8 — Information S1. More detailed description of the main metabolisms encoded by Thioalkalivibrio-related MAGs. Information S2 More detailed description of the main metabolisms encoded by Deltaproteobacterial-related MAGs. (PDF 219 kb) [file 40168_2018_548_MOESM8_ESM.pdf]

Information S1. More detailed description of the main metabolisms encoded by *Thioalkalivibrio*-related MAGs. See also Additional file 6 for the species delineation determined with ANI/ConDNA and Additional file 7 for the presence/absence of selected marker genes (KEGG orthologues).

We recovered 11 MAGs related to *Thioalkalivibrio,* comprising 8 species from which 7 were novel. The potential for aerobic carbon fixation and fermentation under anoxic conditions was evident from the presence of phosphoribulokinase and RubisCo (form I) genes, from L-lactate and formate dehydrogenase (NAD^+ forming) and from pyruvate:ferredoxin oxidoreductase (PFOR) encoding genes, respectively. All MAGs had a gene for sulfide:quinone oxidoreductase (*sqr*) which catalyses sulfide oxidation to elemental sulfur, most MAGs also encoded for an alternative route catalyzed by flavocytochrome c sulfide dehydrogenase (*fccB*). Thiosulfate oxidation via the incomplete Sox pathway, typical for the gammaproteobacterial SOB, was also encoded, all MAGs have a *SoxB* gene, and most MAGs also *SoxXA*, *and SoxYZ*. Capacity for oxidation of sulfite to sulfate was found in all MAGs, both in two steps via adenylylsulfate reductase (*aprAB*) and sulfate adenylyltransferase (*Sat*) and/or directly via sulfite:ferricytochrome-c oxidoreductase (*sor*). Genes for dissimilatory sulfite reductase (*dsrAB*) were absent only in the three most abundant *Tv. spp*. and a gene for thiosulfate/polysulfide reduction (*psrA*) was found only in one of the less abundant MAGs (B1Sed10_47R1). None of the *Thioalkalivibrio* MAGs reconstructed here encoded for thiocyanate degradation.

One MAG from *Tv*. sp. 1 encoded for nitrate reduction to nitrite via a periplasmic nitrate reductase (*napAB*). The MAGs from *Tv* sp. 2 encoded for two types of ammonifying cytochrome *c* nitrite reductases, one homologous to the most common pentaheme type (*nfrA*), another to the octaheme type (*TvNiR*) also found in *Tv. nitratireducens* and *Tv. paradoxus* [1, 2] (Supplementary Figure 12). One of the less abundant *Tv.* MAGs had in addition to *TvNiR* a complete gene set for dissimilatory nitrate reduction to ammonia (*napAB* + *nrfA,* found on different contigs). Two other of the less abundant *Tv.* spp. encoded in addition to *TvNiR* for nitrate reduction to nitrite via nitrate reductase/nitrite oxidoreductase (*nar* genes). Although one also encoded for a nitric oxide reductase (*norC* and *norB*), none of the *Tv.* spp. recovered here encoded for a full set of denitrifying enzymes (absence *nir* and *nos* genes).

Information S2. More detailed description of the main metabolisms encoded by *Deltaproteobacterial*-related MAGs. See also Additional file 6 for the species delineation determined with ANI/ConDNA and Additional file 7 for the presence/absence of selected marker genes (KEGG orthologues).

B1Sed10_16, the highly abundant MAG from the family *Desulfobacteraceae* (order Desulfobacterales, likely genus *Desulfonatronobacter*) encoded for the full dissimilatory reduction of sulfate to sulfide and encoded marker genes for anaerobic reduction of CO_2 via the Wood-Ljungdahl pathway. The MAG did not encode for a CO-methylating acetyl-CoA synthase (EC:2.3.1.169), although the gene was found in two closely related MAGs. An encoded pyruvate:ferredoxin oxidoreductase (PFOR) can link CO_2 reduction to a heterotrophic metabolism or provide intermediates for cell synthesis by reductive carboxylation of acetyl-CoA. The genome encoded also for a formate and L-lactate dehydrogenase, and even 4-hydroxybutyryl-CoA hydratase, which in fermenting Clostridia has a role in γ-aminobutyrate fermentation [3], suggesting the organism can use various electron donors for respiration. The potential for complete oxidation of volatile fatty acids and alcohols, a unique feature for the genus *Desulfonatronobacter* among haloalkaliphilic SRB [4], was encoded by an acetyl-CoA synthase (EC 6.2.1.1). The genome encoded also a fumarate and nitrite reductase (*NrfA*, *NrfH* also found the most closely related MAG).

We recovered 16 MAGs that related to the family *Desulfohalobiaceae* (order *Desulfovibrionales*) in our ribosomal protein tree. One monophyletic clade was related to the genus *Desulfonatronovibrio* and preferred more moderate salinities. A second group included the second dominant lineage of SRB in Bitter-1 and was a new species from the genus *Desulfonatronospira*. A Wood-Ljungdahl pathway was not found in this abundant species, but common among the other *Desulfonatronospira*-related MAGs. Many species encoded for a cytochrome *c* nitrite reductases (*NrfAH*), only a few also for nitrogenase (*nifDHK*), suggesting species-specific potential for dissimilatory nitrite reduction (DNRA) and/or diazotrophic growth. All *Desulfohalobiaceae*-related MAGs encoded a gene for thiosulfate reductase/polysulfide reductase chain A (*PsrA*, *PhsA*) that might be involved in the reducing branch of thiosulfate disproportionation, while the pathway for canonical sulfate reduction was not complete in all MAGs.

References

1. Tikhonova T V., Slutsky A, Antipov AN, Boyko KM, Polyakov KM, Sorokin DY, et al. Molecular and catalytic properties of a novel cytochrome c nitrite reductase from nitrate-reducing haloalkaliphilic sulfur-oxidizing bacterium *Thioalkalivibrio nitratireducens*. Biochim Biophys Acta - Proteins Proteomics. 2006;1764:715–23.

2. Tikhonova T, Tikhonov A, Trofimov A, Polyakov K, Boyko K, Cherkashin E, et al. Comparative structural and functional analysis of two octaheme nitrite reductases from closely related *Thioalkalivibrio* species. FEBS J. 2012;279:4052–61.

3. Berg IA, Kockelkorn D, Ramos-Vera WH, Say RF, Zarzycki J, Hügler M, et al. Autotrophic carbon fixation in archaea. Nat Rev Microbiol. 2010;8:447–60.

4. Sorokin DY, Chernyh NA, Poroshina MN. *Desulfonatronobacter acetoxydans sp. nov*.,: a first acetate-oxidizing, extremely salt-tolerant alkaliphilic SRB from a hypersaline soda lake. Extremophiles. 2015;19:899–907.
